# Supplementary material for: Triboelectric micro-flexure-sensitive fiber electronics
Source: Nat Commun. 2024 Mar 15;15:2374. doi: 10.1038/s41467-024-46516-0 (PMC10943239; doi:10.1038/s41467-024-46516-0)
Supplement: Supplementary file 1 — Supplementary information [file 41467_2024_46516_MOESM1_ESM.pdf]

# Supporting Information

## Triboelectric micro-flexure-sensitive fiber electronics

Shaomei Lin[1], Weifeng Yang[1], Xubin Zhu[1], Yubin Lan[2], Kerui Li[1], Qinghong Zhang[3], Yaogang Li[3], Chengyi Hou[1*], Hongzhi Wang[1*]

Corresponding author: Hongzhi Wang (wanghz@dhu.edu.cn), Chengyi Hou (hcy@dhu.edu.cn)

**This file includes:**

**Supplementary Figures**

**Supplementary Fig. 1** The COMSOL Multiphysics fitting of voltage output for different tribo-gaps in the triboelectric fiber.

**Supplementary Fig. 2** Influence and regulation of interlayer gap on fiber wrinkling amplitude.

**Supplementary Fig. 3** A comprehensive discussion and statistical analysis of crucial parameters relevant to the construction of micro-flexure-sensitive fiber.

**Supplementary Fig. 4** Fabrication process of nanofiber buckling-enabled micro-flexure-sensitive fiber (NB-fiber).

**Supplementary Fig. 5** Relationship between the ability of stress transfer and molecular structural formula of the inner tribo-layer EVA and the out tribo-layer TPU NFM.

**Supplementary Fig. 6** Fabrication principles and performance characterization of ionogel electrodes.

**Supplementary Fig. 7** Supplementary Fig. 7 The electrical properties of Ionogels with varying AAm content.

**Supplementary Fig. 8** Uniformity demonstration of 50-meter continuous production process samples.

**Supplementary Fig. 9** The wrinkle details of NB-fiber.

**Supplementary Fig. 10** Strain and modulus variations of shape memory polymers (EVA) at different temperatures.

**Supplementary Fig. 11** The Anti-interference test of different electrodes on electrical signals.

**Supplementary Fig. 12** Working mechanism and influencing factors discussion of NB-fiber.

**Supplementary Fig. 13** The sensitivity of NB-fiber to small-angle bending.

**Supplementary Fig. 14** Mechanical fatigue testing of NB-Fiber.

**Supplementary Fig. 15** Mechanical fatigue testing of NB-fiber using three-point bending mode.

**Supplementary Fig. 16** The impact of 10,000 long cycles on triboelectric voltage and gel conductivity.

**Supplementary Fig. 17** The manufacturing process and physical photos of the muscle force monitoring cuff.
**Supplementary Fig. 18** Detection and analysis of the human pulse.
**Supplementary Fig. 19** Different forearm positions for muscle force testing.
**Supplementary Fig. 20** Monitoring flexor carpi radialis muscle deformation under different grip forces.
**Supplementary Fig. 21** NB-fiber Micro-Deformation Monitoring Applications.
**Supplementary Fig. 22** Comparative study of vital capacity and respiratory rate tests.
**Supplementary Fig. 23** Weaving process and details of NB-Textile.
**Supplementary Fig. 24** Breathability and moisture permeability performance of NB-textile.
**Supplementary Fig. 25** Triboelectric performance of NB-fiber when exposed to different states of human skin (dry, secreting liquid sweat, oily).
**Supplementary Fig. 26** Water resistance test of NB-textile.
**Supplementary Fig. 27** Circuitry and functional analysis of the multi-channel sensing system.
**Supplementary Fig. 28** Application of NB-fiber for bent gesture recognition.
**Supplementary Fig. 29** Hand press on fabric induces curvature change illustration.
**Supplementary Fig. 30** The impact of different pressures and contact areas on NB-fiber triboelectric voltage.
**Supplementary Fig. 31** Pressure mapping of various gestures and different doll sizes on NB-Textile.
**Supplementary Fig. 32** Demonstration of muscle force mapping software under different hand gesture states.
**Supplementary Fig. 33** The dataset of different hand gesture actions demonstrates the repeatability and stability of the monitoring system.

**Supplementary Tables**

**Supplementary Table 1** Compilation of literature on triboelectric systems for biomechanical bending sensing.
**Supplementary Table 2** Summary of testing details and data results from the cited reference.
**Supplementary Tables 3** Summary of strain sensing yarns: sensing mechanisms, electrical output, scalable manufacturing, recycling, and applications.

**Supplementary Notes**

**Supplementary Note 1** Regulating the amplitude of fiber wrinkling in the model and its correlation with flexure sensitivity.
**Supplementary Note 2** Synthesis of ionogel electrodes.
**Supplementary Note 3** Analyzing the reasons for insensitivity to bending in fermat spiral spinning and built-in spiral.
**Supplementary Note 4** Comprehensive evaluation of flexure performance using curvature.
**Supplementary Note 5** Flexure factor: defining and quantifying bending sensitivity.
**Supplementary Note 6** Calculation method and analysis of augmentation index.

## Supplementary Figures

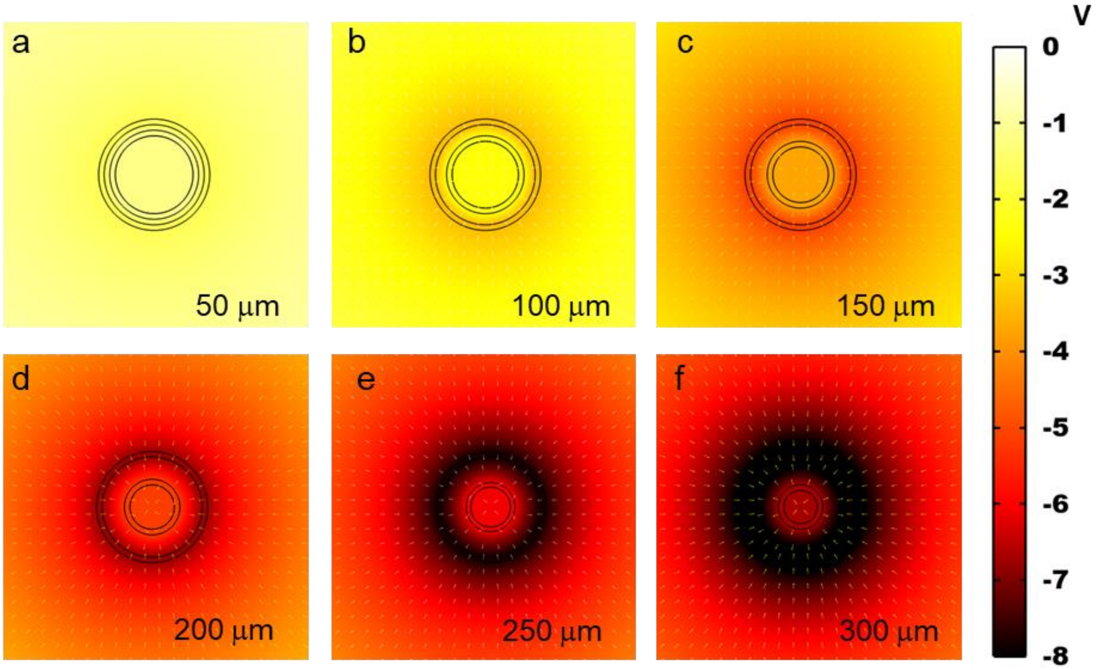


**Supplementary Fig. 1 The COMSOL Multiphysics fitting of voltage output for different tribo-gaps in the triboelectric fiber. a–f** Correspond to voltage output for gap sizes of 50 μm, 100 μm, 150 μm, 200 μm, 250 μm, and 300 μm, respectively.

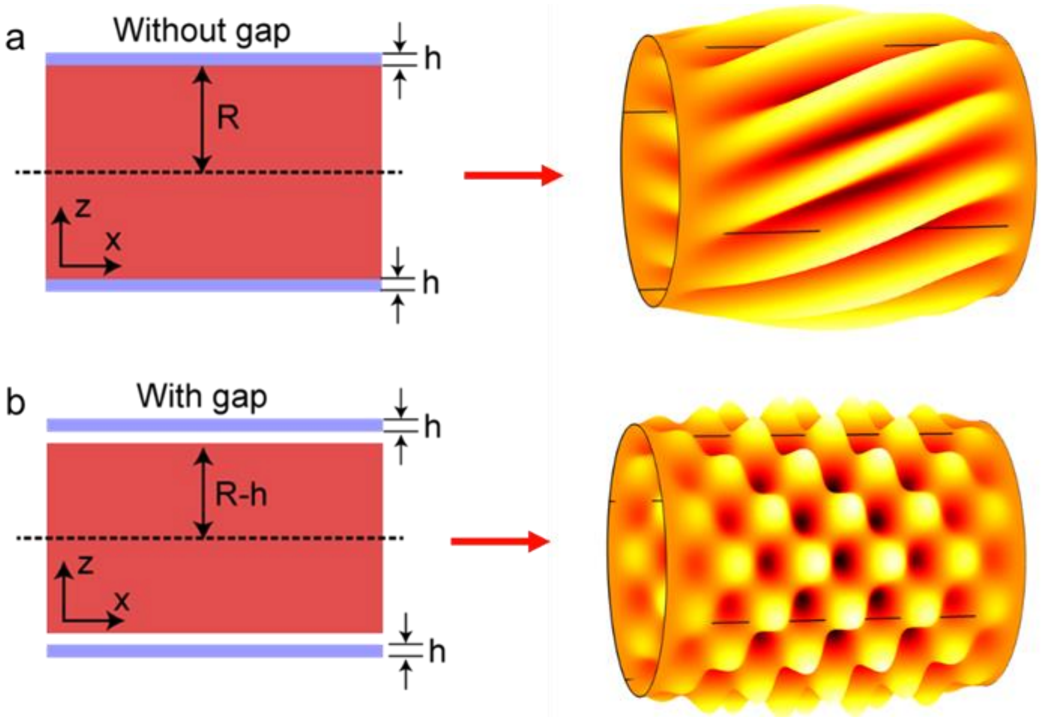


**Supplementary Fig. 2 The illustration depicts how the interlayer gap influences and regulates fiber wrinkling amplitude in the schematic model. a** There is no interlayer gap between the sheath and core. **b** An interlayer gap is present between the sheath and core. This study underscores the capability to control and select unstable modes by adjusting the gap size.

Specifically, when there is minimal or no gap between the core and sheath before structural buckling occurs, it results in a stable sinusoidal axisymmetric deformation. In contrast, a larger interlayer gap induces a defect-sensitive non-axisymmetric "diamond-like pattern" mode during buckling. [Ref.: "On axisymmetric/diamond-like mode transitions in axially compressed core-shell cylinders. J. Mech. Phys. Solids 94, 68-87."]

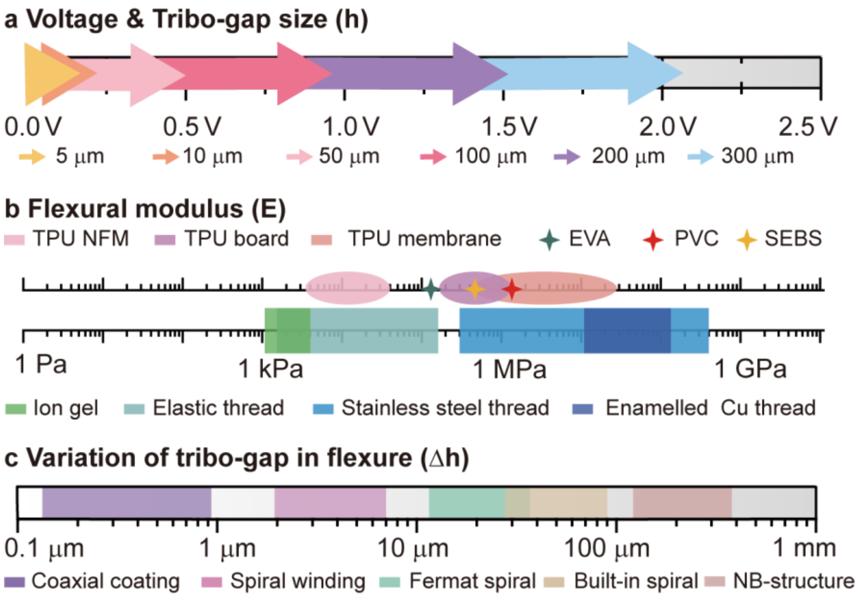


**Supplementary Fig. 3 A comprehensive discussion and statistical analysis of crucial parameters relevant to the construction of micro-flexure-sensitive fiber. a** Relationship between the output voltage of triboelectric fibers and tribo-gap size (h). The output voltage demonstrates an increasing trend with the increment of h. **b** Statistics of the flexure modulus (E) for the micro-flexure-sensitive fiber's outer, inner, and electrode core layers. Utilizing low-modulus TPU NFM and ionogels facilitates substantial deformations and notable variations in tribo-gap during the deformation process. **c** Statistical analysis of the variable tribo-gap range in several state-of-the-art fiber structures during bending deformations. The built-in spiral structure exhibits a significant tribo-gap. However, the limited flexure-induced topological deformations and the relatively similar moduli of the sheath-core materials result in less pronounced tribo-gap variation. In contrast, the NB-structure, incorporating low-modulus TPU NFM in the sheath layer, enables a more pronounced separation from EVA under the influence of external forces.

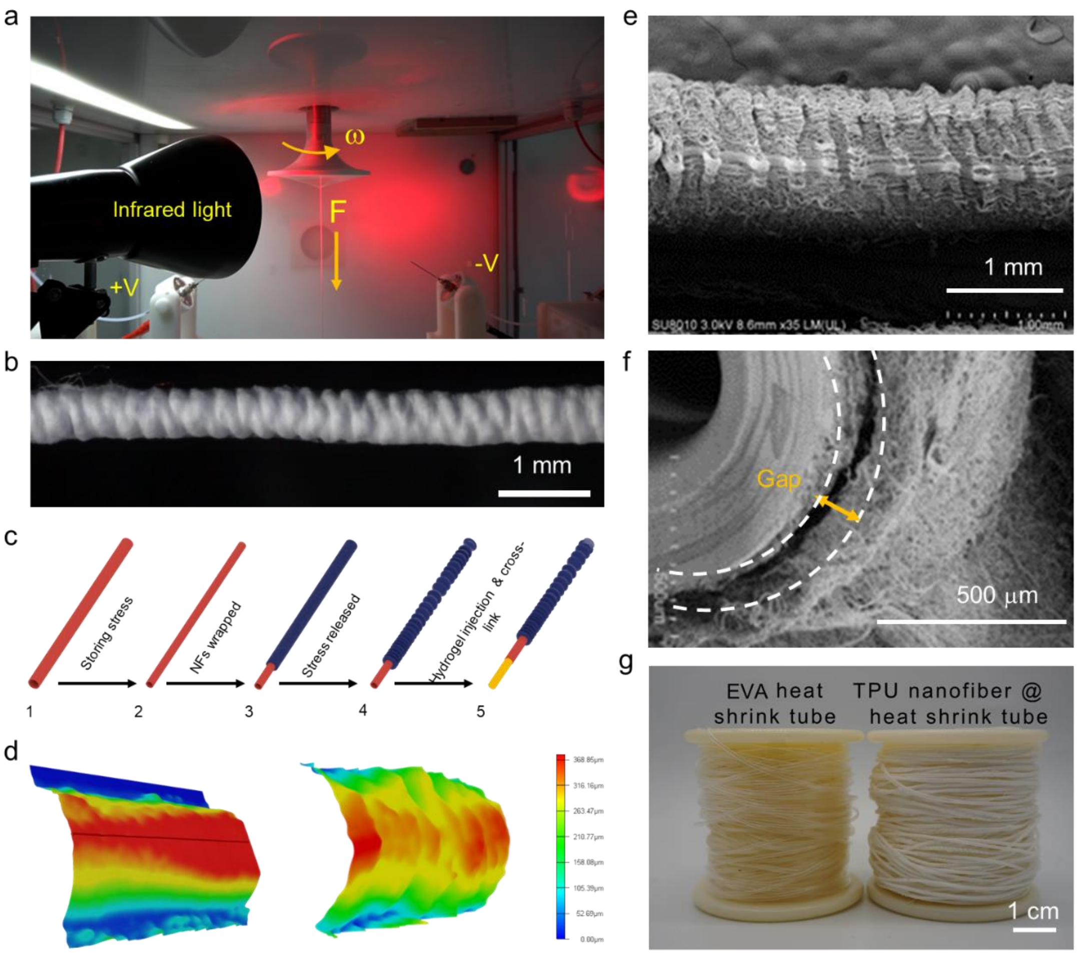


**Supplementary Fig. 4 Fabrication process of nanofiber buckling-enabled micro-flexure-sensitive fiber (NB-fiber). a** Controlled manipulation of the electrostatic, velocity, and stress fields in Fermat electrospinning to achieve ordered twisting of TPU nanofibers on EVA fiber. Infrared light is applied to facilitate the curing of TPU nanofibers. **b** External view of the NB-fiber. The fiber obtained from Fermat spinning is infused with ionogel precursor and heated at 60°C for one hour to release stress and solidify the electrodes, resulting in a fiber structure with nanofiber buckling. **c** Schematic representation of the surface morphology at each step of NB-fiber fabrication. **d** Surface roughness of NB-fiber before and after annealing, analyzed using a 3D optical profilometer. **e** Scanning electron microscopy image of NB-fiber. **f** Electron fiber microscopy cross-sectional image of NB-fiber. **g** Comparative images of EVA fiber and NB-fiber.

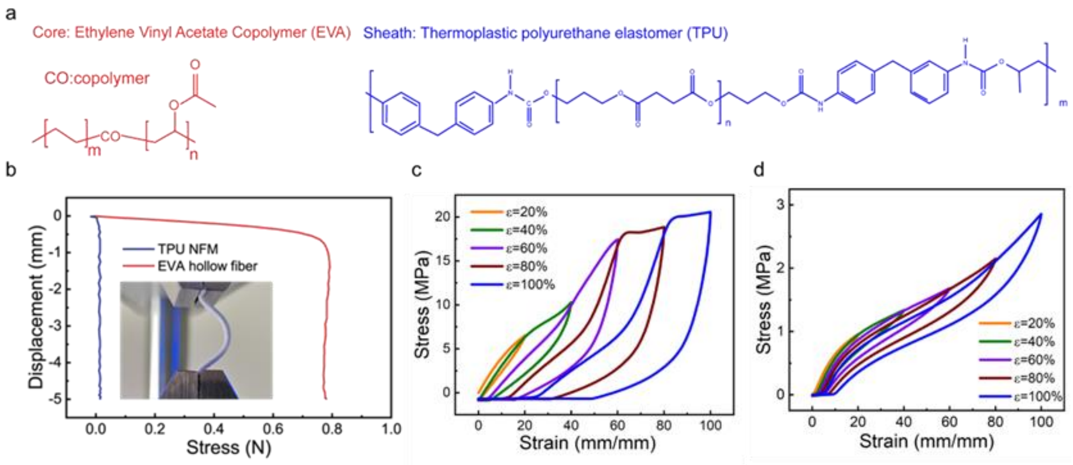


**Supplementary Fig. 5 Relationship between the ability of stress transfer and molecular structural formula of the inner tribo-layer EVA and the out tribo-layer TPU NFM. a** Molecular structural formulas of EVA and TPU. **b** Stress requirements for achieving the same behavioral displacement in TPU NFM and EVA fibers. This indicates that NB-fibers with TPU NFM exhibit significant deformation under flexural stress. **c** Tensile hysteresis curve of EVA fibers. d, Tensile hysteresis curve of TPU@EVA fibers.

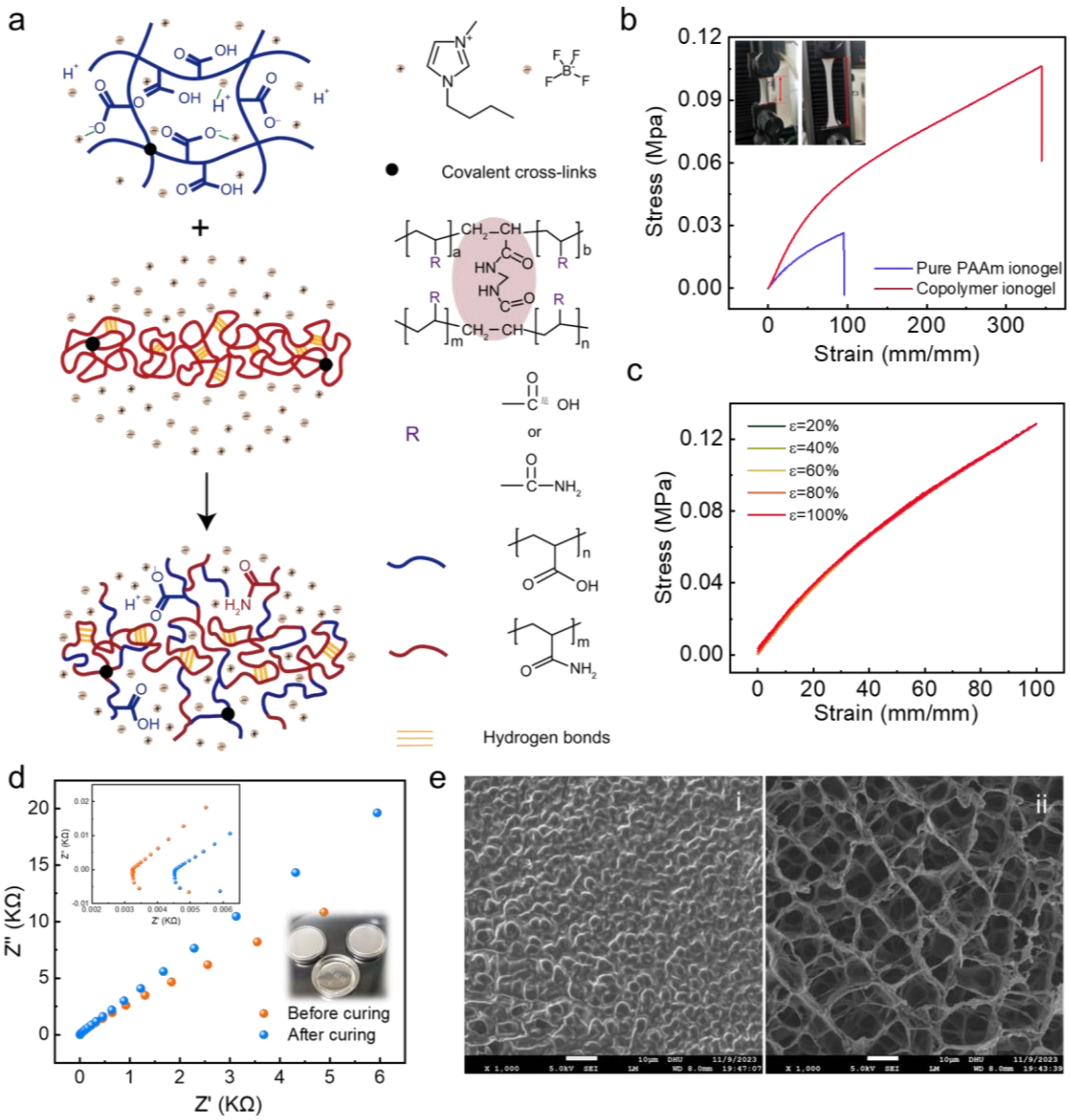


**Supplementary Fig. 6 Fabrication principles and performance characterization of ionogel electrodes. a** To achieve highly tough and stretchable ionogels, a random copolymerization of two typical monomers is performed using polymers with different solubilities in the 1-Hexyl-3-methylimidazolium tetrafluoroborate ionic liquid. Acrylamide and acrylic acid are copolymerized, resulting in a macroscopically uniform covalent network with in-situ phase separation: a hydrogen-bond-rich polymer phase that dissipates energy and toughens the ionogel and an elastic solvent-rich phase capable of undergoing large strains. **b** A comparison of the tensile fracture behavior between dual-component copolymer ionogels and pure PAAm ionogels. The copolymer ionogels exhibit significantly higher strength and strain than pure PAAm ionogels. **c** Tensile cyclic tests of the copolymer ionogels, demonstrating their excellent recovery properties. **d** Electrical conductivity before and after the solidification of the ionogel. **e** Scanning electron microscope images of the surface morphology of the ionogel (i) and the polymer skeleton (ii).

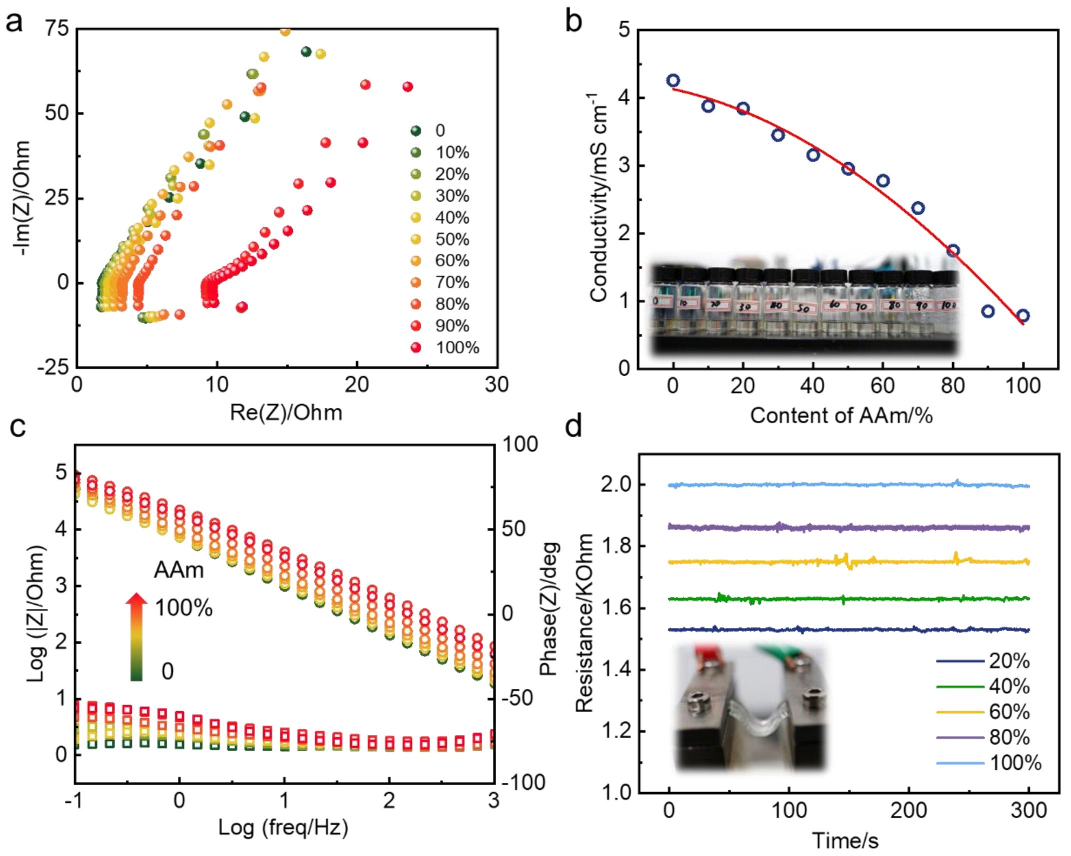


**Supplementary Fig. 7 The electrical properties of Ionogels with varying AAm content. a** Nyquist impedance plots for Ionogels at different AAm ratios. **b** Statistical analysis of electrical conductivity. **c** Bode plots. **d** Resistance testing during dynamic bending processes.

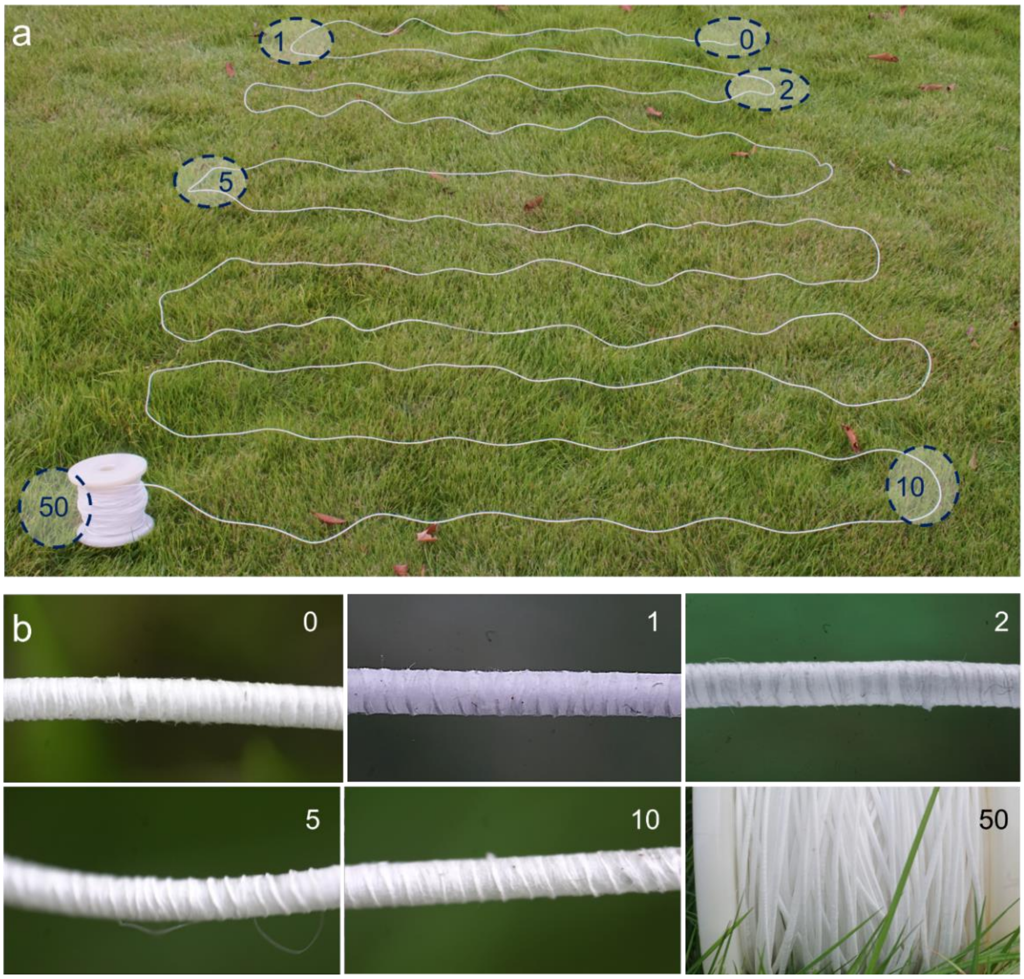


**Supplementary Fig. 8 Uniformity demonstration of 50-meter continuous production process samples. a** macroscopic display and **b** corresponding local magnified photographs.

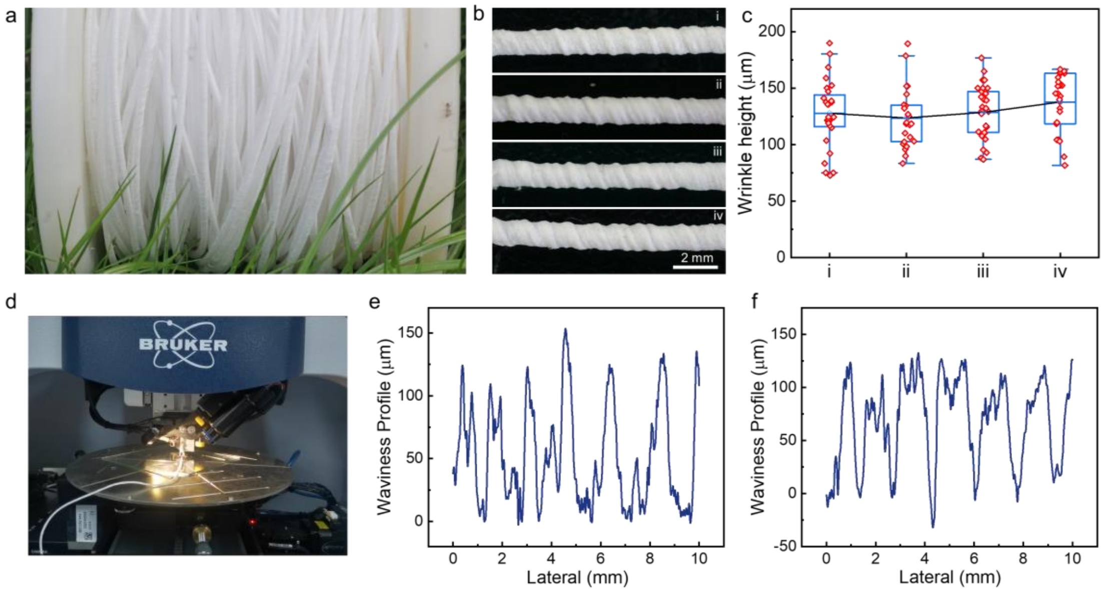


**Supplementary Fig. 9 The wrinkle details of NB-fiber. a** Macroscopic view of wrinkles on a 50-meter-long NB-fiber. **b** Microscopic enlargement of a randomly selected 2-centimeter-long NB-fiber (4 randomly selected sections of the fiber are labeled as i-iv). **c** Corresponding

statistical analysis of wrinkle heights. **d** Detailed images from roughness measurements obtained using Dektak XT stylus profiler. **e-f** Surface roughness scan waveforms of NB-fiber at different locations.

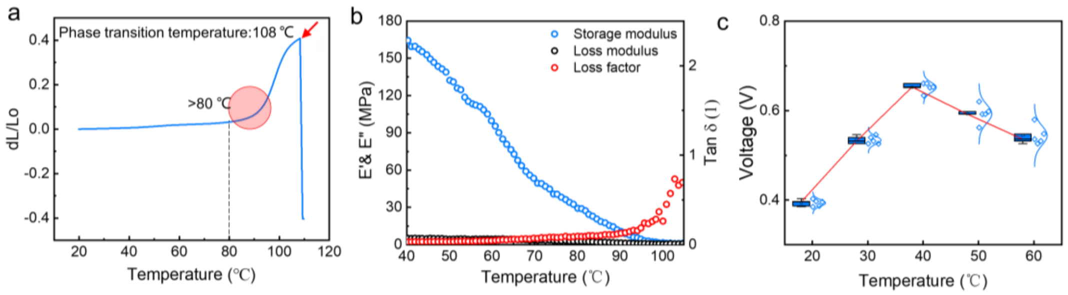


**Supplementary Fig. 10 Strain and modulus variations of shape memory polymers (EVA) at different temperatures. a** Thermomechanical analysis of EVA demonstrates deformation initiation at 80°C. **b** Dynamic mechanical analysis of the material's viscoelastic behavior, including storage modulus, loss modulus, and tan delta values. **c** The triboelectric voltage of NB-fiber at different temperatures.

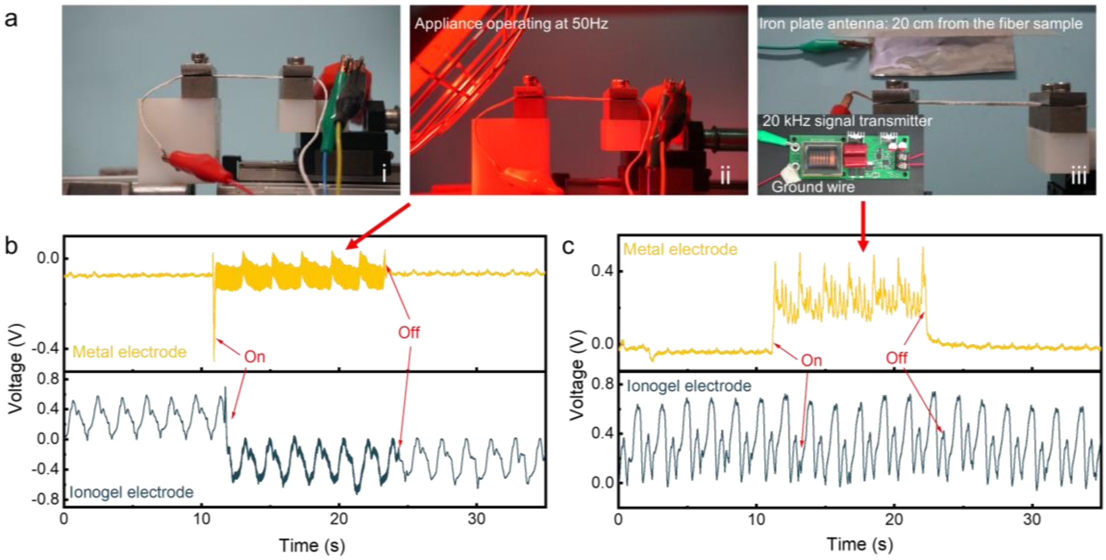


**Supplementary Fig. 11 The Anti-interference test of different electrodes on electrical signals. a** Various test scenarios: i. No additional electromagnetic sources nearby. ii. Operation of electrical appliances at a frequency of 50 Hz. iii. 20 kHz electric field generator. **b** Performance of metal electrodes and ionogel electrodes under operation of electrical appliances. **c** Performance of metal electrodes and ionogel electrodes under a 20 kHz electric field.

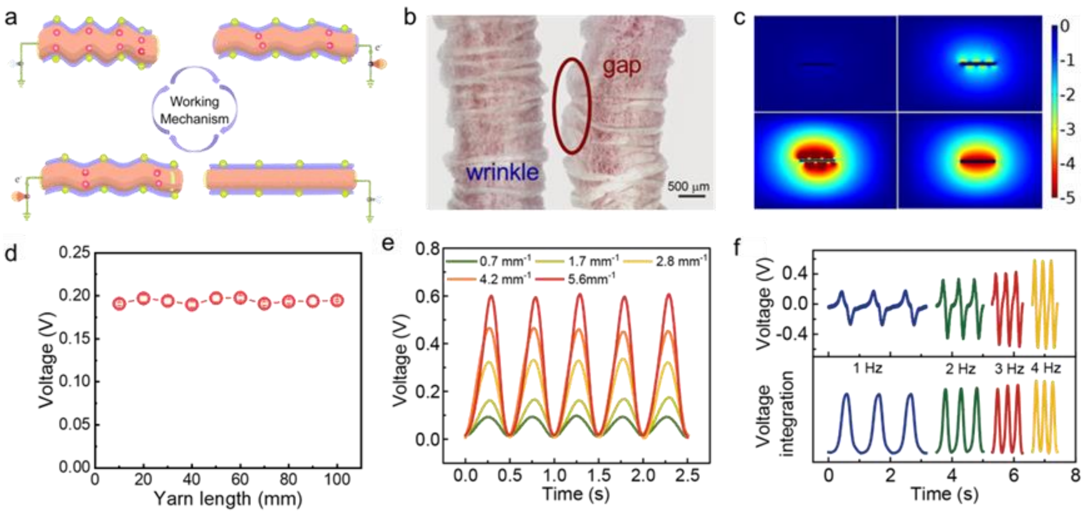

**Supplementary Fig. 12 Working mechanism and influencing factors discussion of NB-fiber. a** Schematic representation of surface roughness and charge distribution on the NB-fiber during the bending-recovery process. **b** Structure of the gap between the TPU nanofiber sheath and EVA fiber core in the NB-fiber. Red ink is introduced in the ionogel to enhance the gap structure's visualization. **c** Relationship between surface roughness and potential distribution simulated using COMSOL Multiphysics. **d** Triboelectric voltage of different lengths of NB-fiber under a bending curvature of 2 mm$^{-1}$. **e** Electrical performance output of a 5 cm long NB-fiber at different curvatures. The voltage exhibits a linear relationship with increasing curvature. The NB-fiber reaches an output voltage 0.6V at a curvature of 5.6 mm$^{-1}$. **f** Influence of bending frequency on the electrical output of the NB-fiber. The output voltage increases with increasing frequency. The voltage integral is utilized to eliminate the effect of frequency on the output, resulting in peak values of the voltage integral that remain constant at different frequencies.

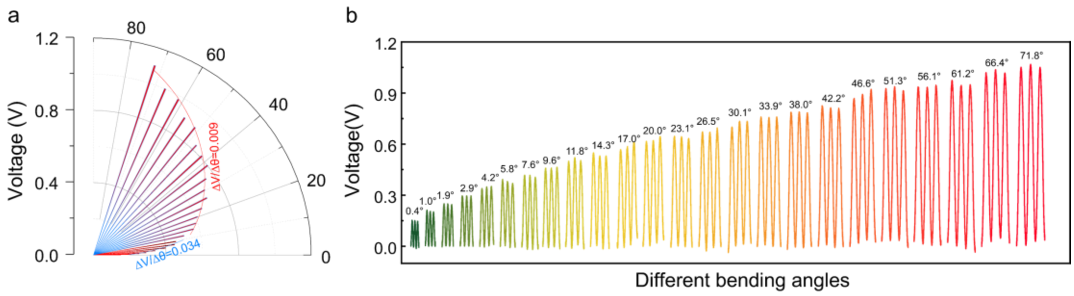

**Supplementary Fig. 13 The sensitivity of NB-fiber to small-angle bending. a** NB-fiber exhibits a higher bending response capability at angles below 10° compared to angles exceeding 10° (indicated by the slope), demonstrating its sensitivity to minor angular deformations. **b** The specific electrical signal waveforms of NB-fiber at angles ranging from 0.4° to 71°.

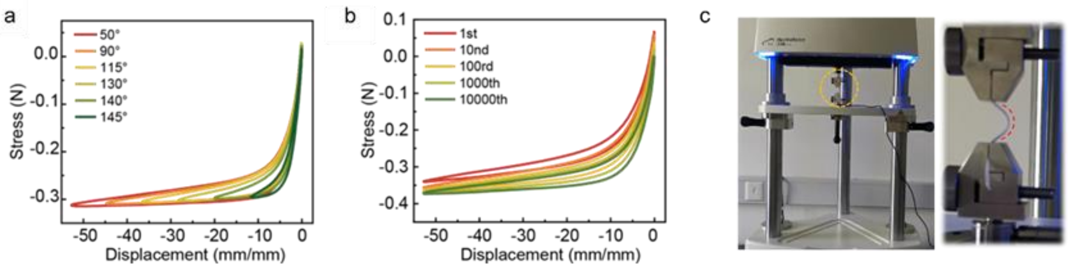


**Supplementary Fig. 14 Mechanical fatigue testing of NB-Fiber. a** Bending cyclic curves of the NB-fiber at different bending angles. Notably, 145° exceeds the range of motion for most human joints. **b** Ten thousand bending cycles at 145°. After ten thousand cycles, the overall curve slightly shifts downward, approximately 14% compared to the initial state. **c** The testing was conducted using the Electro Force 3230 equipment, and the sample was appropriately clamped to ensure accurate measurements.

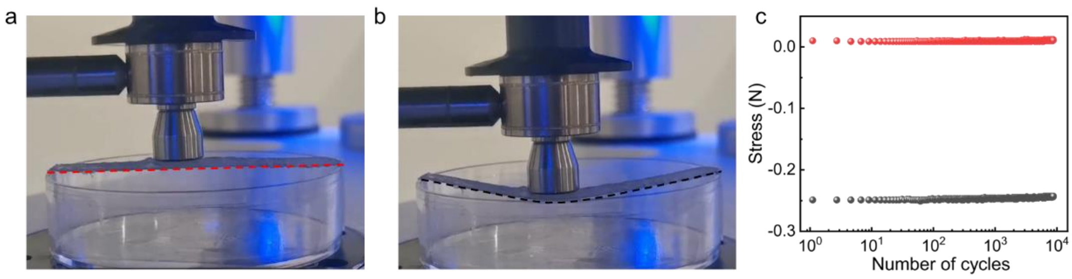


**Supplementary Fig. 15 Mechanical fatigue testing of NB-fiber using three-point bending mode. a** Initial state. **b** Flexure state. **c** Stress distribution at the start position during 10,000 cycles.

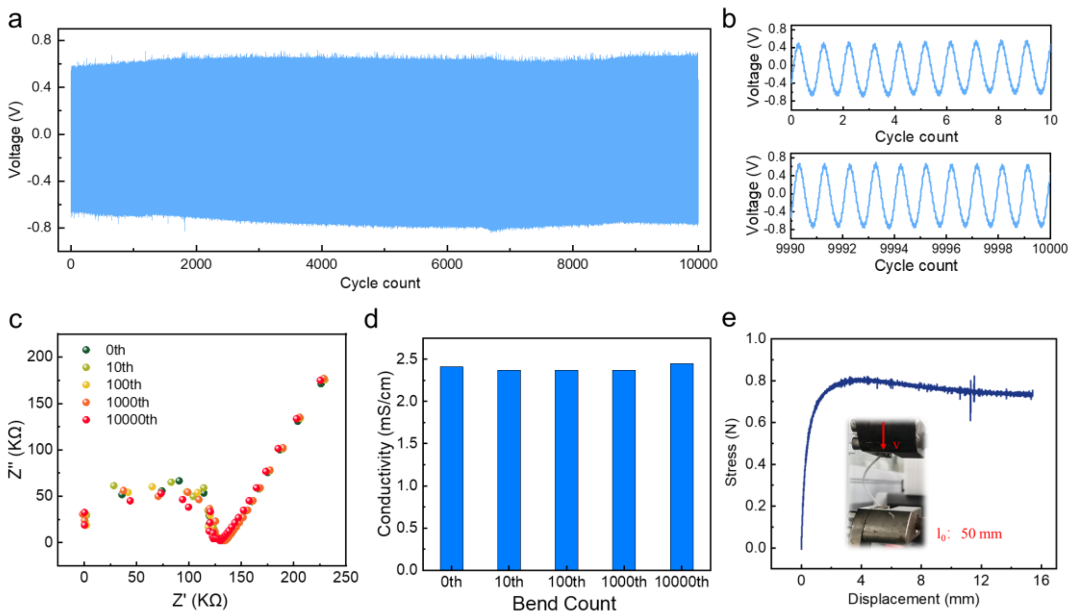

**Supplementary Fig. 16 The impact of 10,000 long cycles on triboelectric voltage and gel conductivity. a-b** Electrical properties of NB-fiber in bending fatigue tests. **c-d** The conductivity of the NB-fiber after 1, 10, 100, 1000, and 10,000 bending cycles. **e** Characterization of mechanical stress conditions on NB-fiber during electrical testing phases described in Figures a-d.

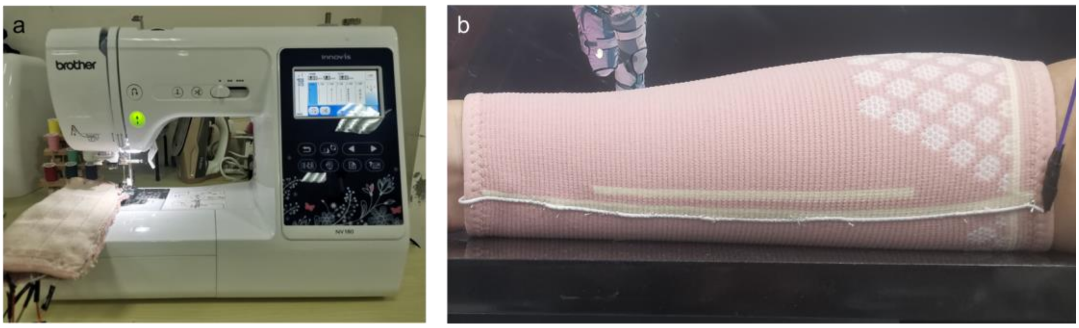


**Supplementary Fig. 17 The manufacturing process and physical photos of the muscle force monitoring cuff. a** Use a Brother NV180 sewing machine to stitch the NB-fiber onto a commercial sleeve, and add a physical connector for connecting the PCB board. **b** Translation of the finished photo of the muscle force monitoring sleeve.

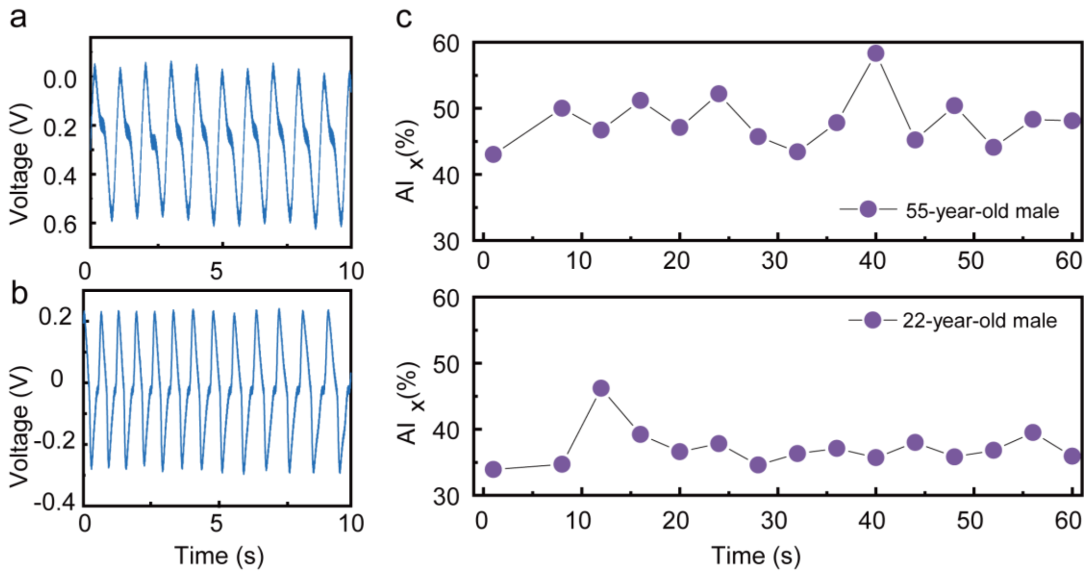


**Supplementary Fig. 18 Detection and analysis of the human pulse. a** Pulse monitoring of a 55-year-old male. **b** Pulse monitoring of a 22-year-old male. **c** Comparison of aix indices among different age groups.

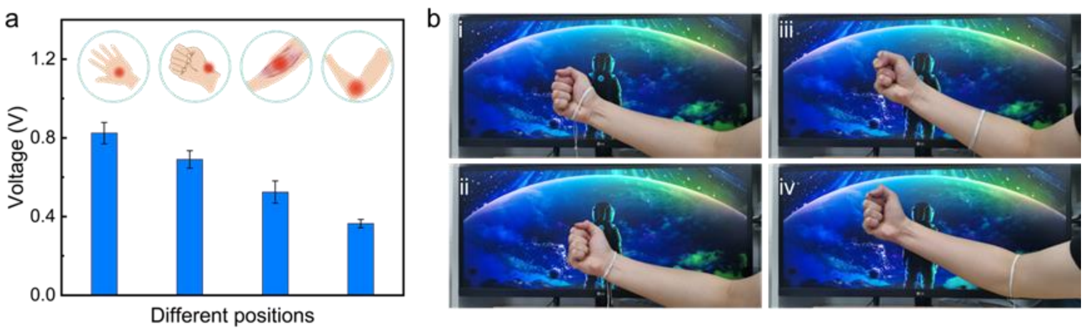


**Supplementary Fig. 19 Different forearm positions for muscle force testing. a** Muscle deformation monitoring at various locations such as dorsum of the hand, wrist, forearm, and elbow, under the same grip force. **b** Placement of NB-fiber during the testing.

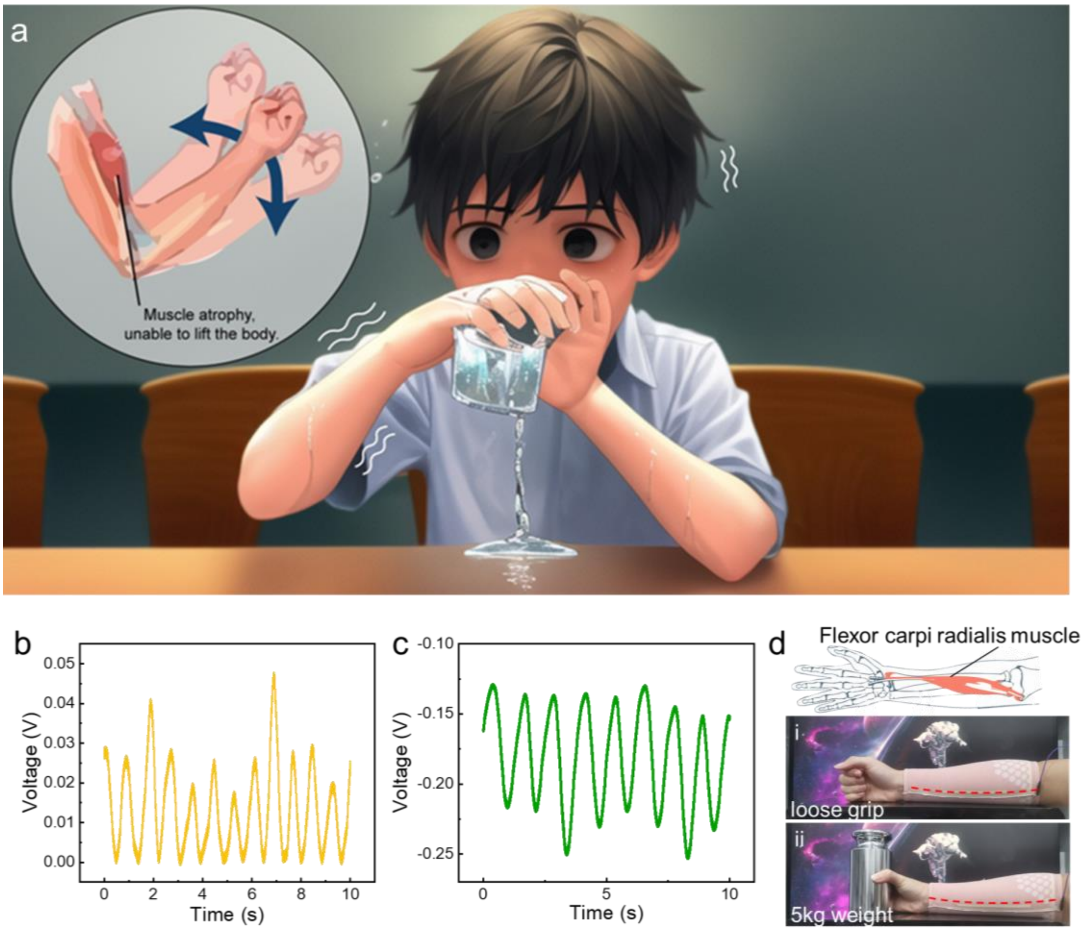


**Supplementary Fig. 20 Monitoring flexor carpi radialis muscle deformation under different grip forces. a** A scene showing a muscle atrophy patient struggling to pick up a glass of water due to limited muscle strength. **b** Muscle deformation signal observed during loose grip. **c** Muscle deformation signal recorded while lifting a 5 kg weight. **d** Detailed description of the testing procedure employed during the experimental process.

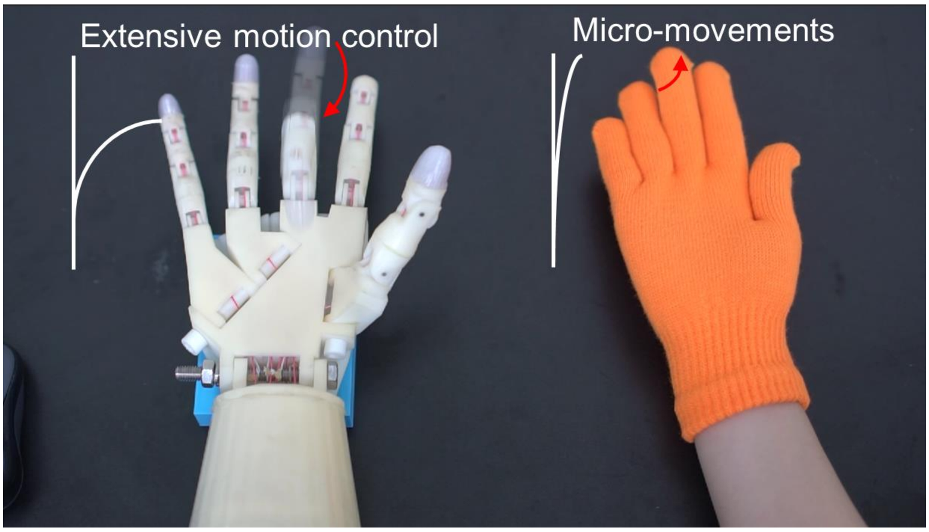


**Supplementary Fig. 21 NB-fiber Micro-Deformation Monitoring Applications.** NB-fiber can help patients with motor neurological disorders that impact hand and arm movement to achieve motion control of robotic prosthetics as an assistive solution.

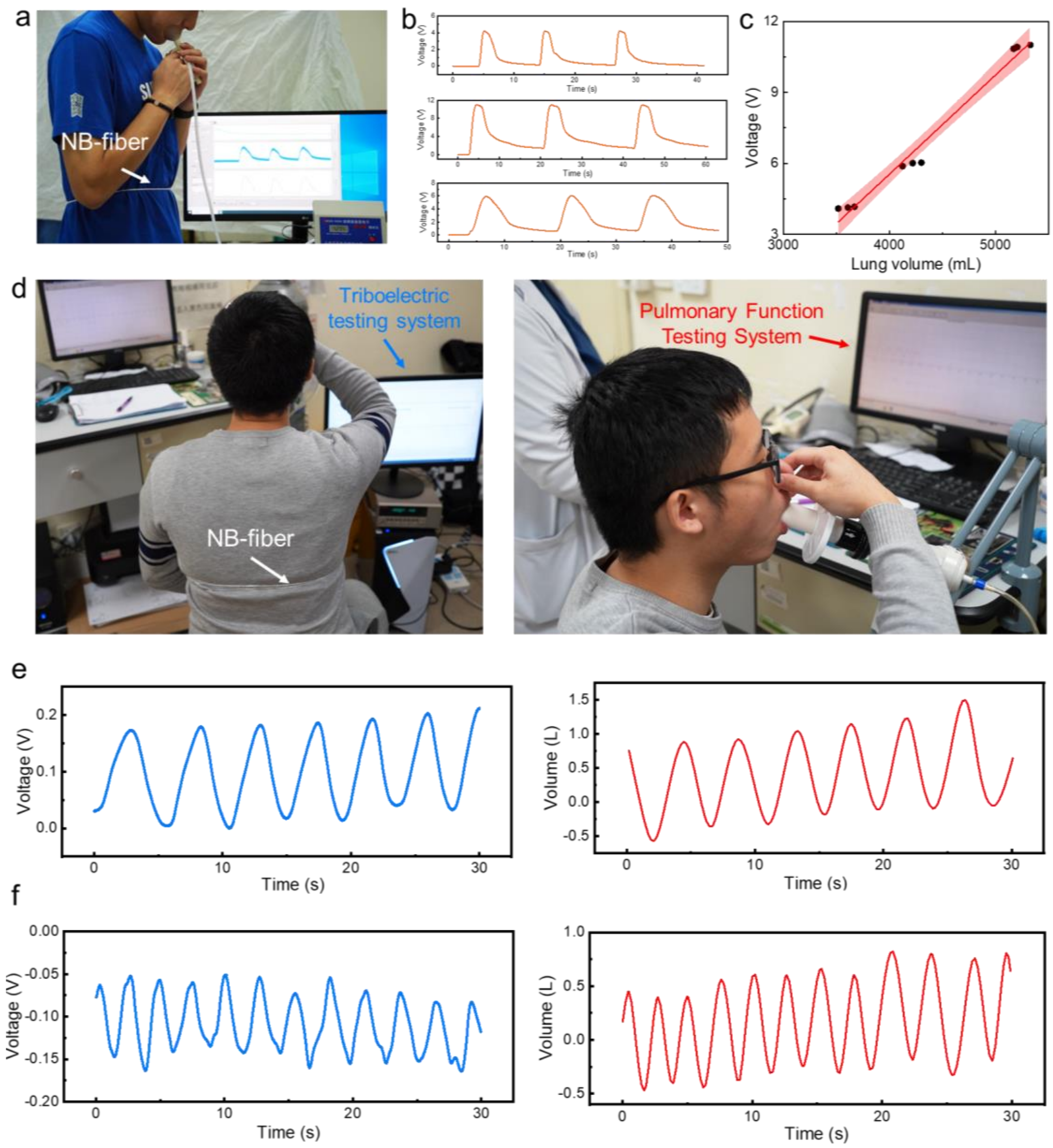


**Supplementary Fig. 22 Comparative study of vital capacity and respiratory rate tests. a** Vital capacity was simultaneously measured using NB-fiber and a medical pulmonary function testing device. **b** NB-fiber signal curves corresponding to different pulmonary capacities. **c** Confidence intervals for NB-fiber signals and their corresponding pulmonary function test values. **d** Comparative measurement of respiratory rate using NB-fiber and a medical pulmonary function testing device simultaneously. **e** Comparison of respiratory signal curves for a 20-year-old male. **f** Comparison of respiratory signal curves for a 26-year-old female. (Left: Triboelectric testing system, Right: Pulmonary function testing system).

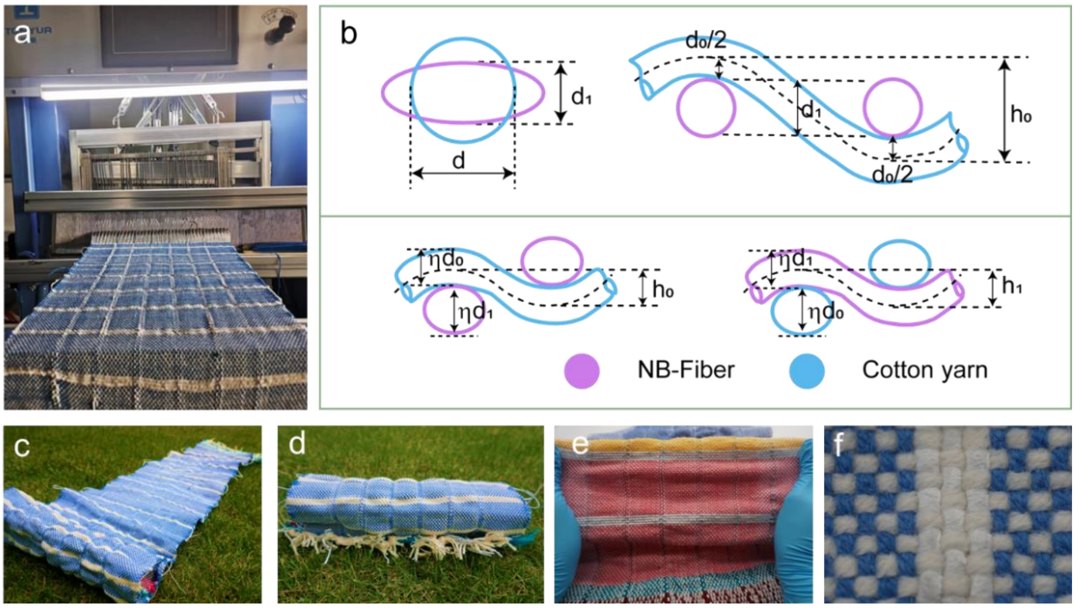


**Supplementary Fig. 23 Weaving process and details of NB-textile. a** The NB-fiber and cotton thread are blended and woven using a commercial weaving machine. **b** Comparison between the ideal and actual cross-sectional states of the NB-fiber and cotton thread during the weaving process. **c** Actual photograph of the NB-textile, with a length of approximately 1.5 m and a width of 0.3 m. **d** Curvature of the NB-textile after weaving. **e** Tensile behavior of the NB-textile, showing minimal deformation under tension. **f** Post-weaving characterization of the NB-structure, demonstrating the well-preserved integrity of the NB-structure.

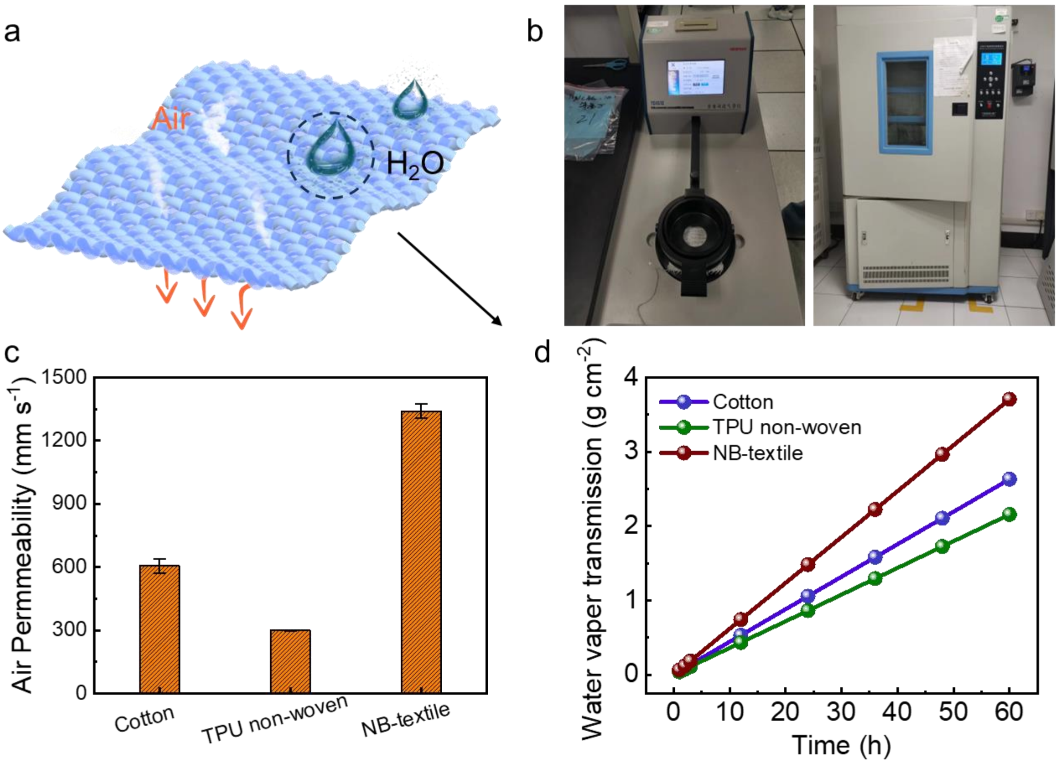


**Supplementary Fig. 24 Breathability and moisture permeability performance of NB-textile. a** The woven structure of NB-textile allows for unhindered airflow and water vapor

transmission. **b** Testing equipment for air permeability (left) and moisture permeability (right). **c** Air permeability testing of commercial cotton fabric, TPU nonwoven fabric, and NB-textile. **d** Moisture permeability testing of commercial cotton fabric, TPU nonwoven fabric, and NB-textile.

The air permeability of the textile was assessed using an air permeability tester (YG461G, Wenzhou Fangyuan Instrument Co., Ltd., China) by the standard GB/T 24218.15-2018. The water vapor transmission rate was measured using a fabric moisture permeability testing apparatus (YG601H, Ningbo Textile Instrument Factory, China) following the GB/T 12704.1-2009 standard.

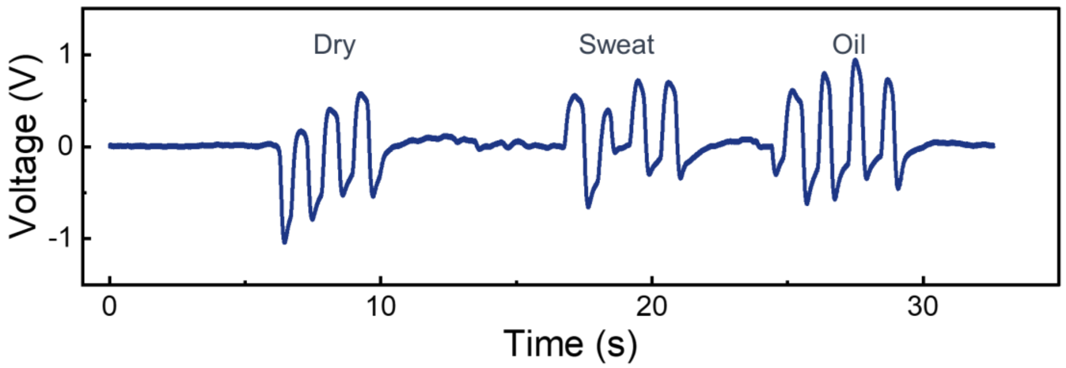


**Supplementary Fig. 25 Triboelectric performance of NB-fiber when exposed to different states of human skin (dry, secreting liquid sweat, oily).**

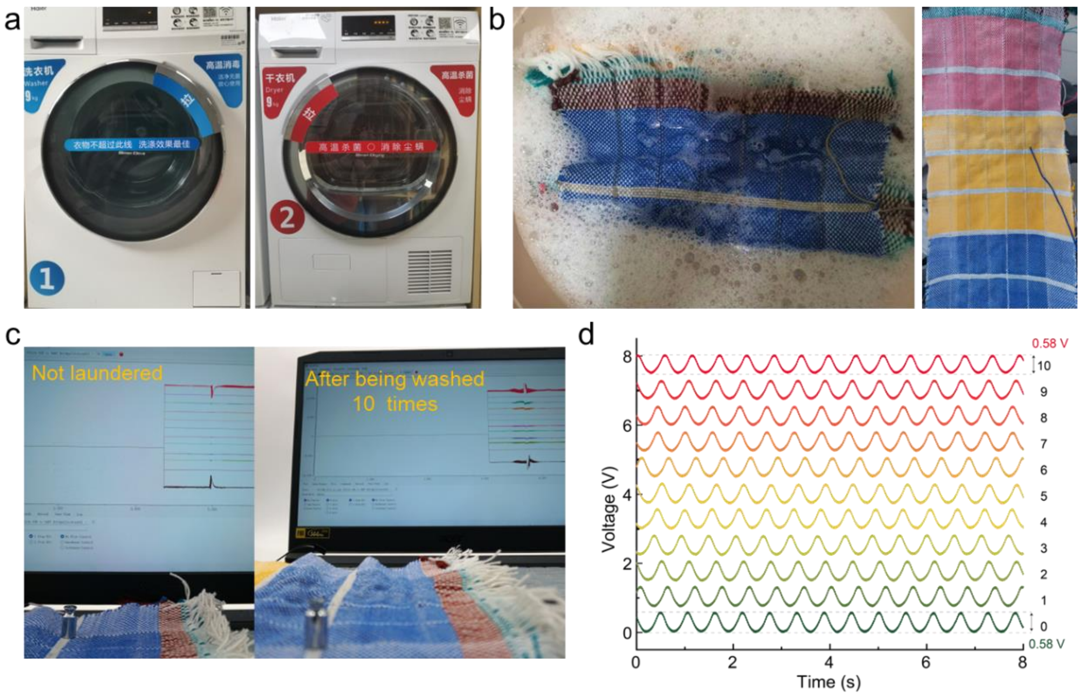


**Supplementary Fig. 26 Water resistance test of NB-textile. a** Commercial washing machine and dryer used for the test. **b** Images showing the samples before and after the water washing and drying. **c** Comparison of sensing performance between samples before and after water washing, using a 10g weight. **d** The triboelectric performance test of NB-fiber after 10 water washes, with a length of 10 cm and a bending angle of 30°.

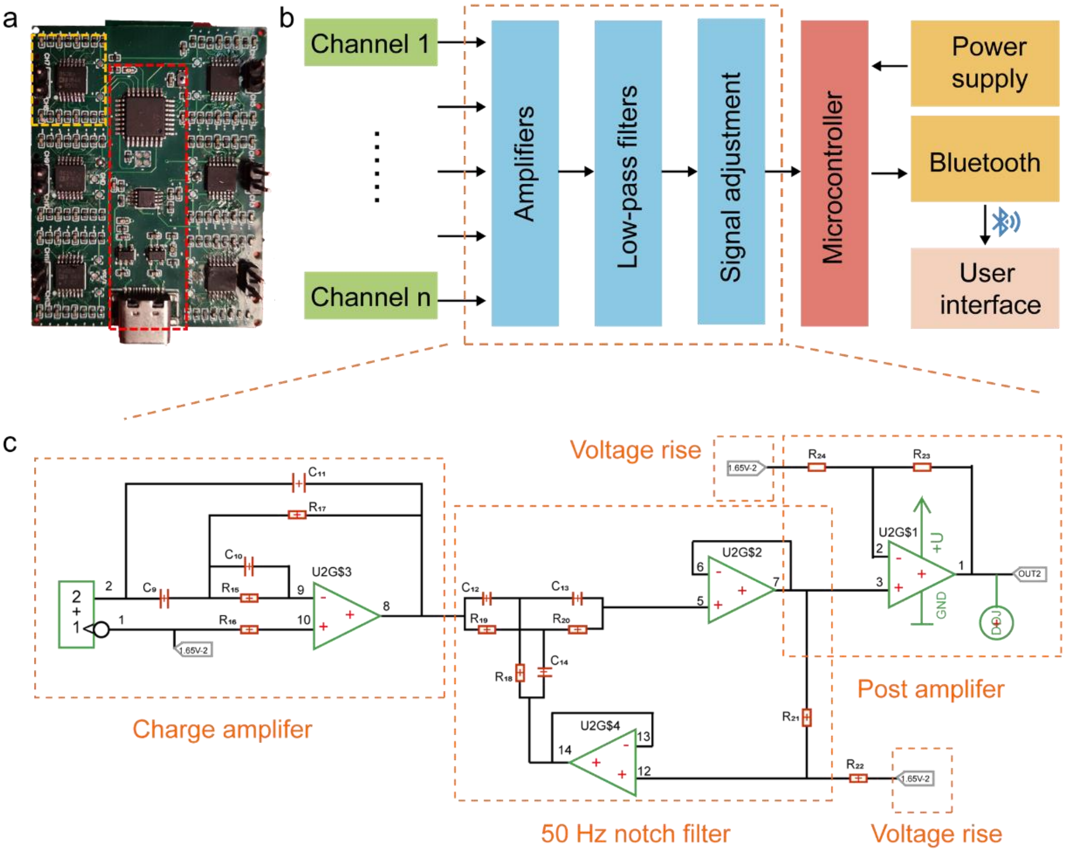


**Supplementary Fig. 27 Circuitry and functional analysis of the multi-channel sensing system. a** Physical layout of the 12-channel circuit board. **b** Various functional modules integrated into the circuit board. **c** Sensing signal processing circuit.

Supplementary Fig. 26 provides an in-depth analysis of the circuitry and functionality of the multi-channel sensing system. The integrated PCB facilitates signal processing, wireless transmission, and accurate gesture recognition, making it a crucial component of the overall system. The analog signals generated by all signal components were wirelessly transmitted to the terminal display through a printed circuit board (PCB) worn on the wrist, as illustrated in Supplementary Fig. 26a. The PCB integrated multiple functions, including signal conditioning, processing, and wireless transmission, as depicted in Supplementary Fig. 26b. The yellow dotted boxes represent integrated circuit components such as analog signal amplifiers, low-pass filters, and software-based adjustments. By amplifying the collected signal, the low-pass filter effectively eliminated interference signals and environmental noise, ensuring the accurate expression of gesture information through the final analog output of the NB-fiber sensor. The red dashed boxes correspond to the power module, microcontroller with computing and serial communication capabilities, and wireless transceiver, which collectively enable the signal

collection, processing, and wireless transmission to the terminal. In the processing of the triboelectric signal, the following steps are involved:

(i) When the NB-fiber sensor is bent, it produces a triboelectric charge signal, which is converted into a voltage, and amplified using a charge amplifier.

(ii) To address power frequency interference in the output signal, a 50 Hz notch filter is employed.

(iii) A voltage-proportional amplifier is included to adjust the output amplitude and gain.

(iv)In order to use the voltage signal for analog-to-digital conversion by the microcontroller unit (MCU), the signal must be raised to the range of the ADC acquisition (0~3.3 V).

(v) The digital signal is transmitted to the personal mobile terminal via Bluetooth transmission protocol through the MCU, while the analog electric signal is output to control the actuators.

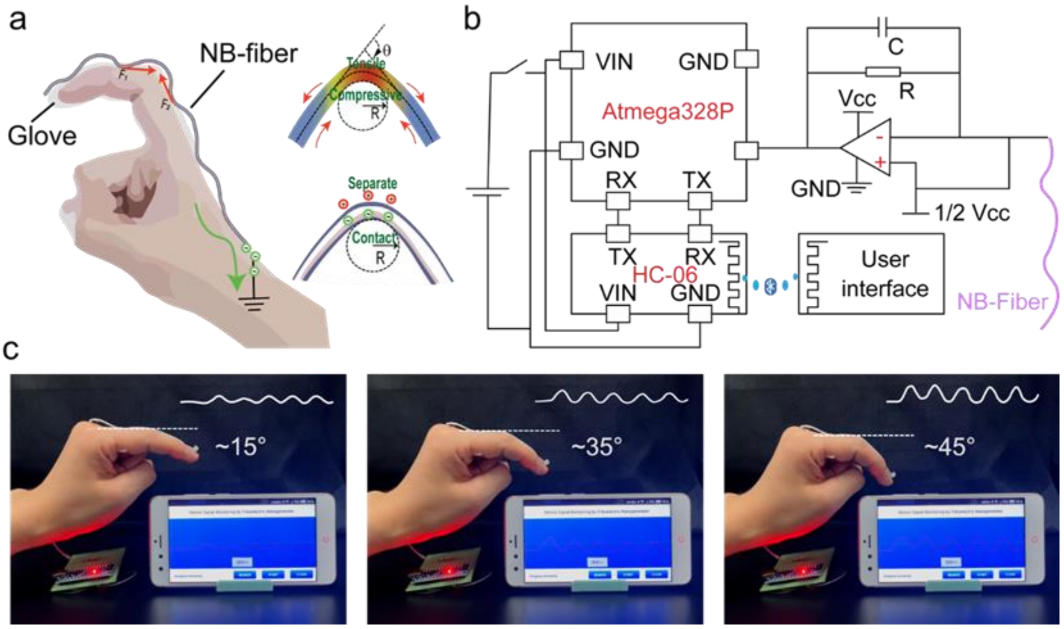


**Supplementary Fig. 28 Application of NB-fiber for bent gesture recognition. a** Force distribution and stress-electrode charge distribution of the NB-fiber during finger bending. **b** Bluetooth wireless transmission circuit system is implemented to facilitate the seamless transfer of data.. **c** Voltage waveforms of the finger at bending angles of 15°, 35°, and 45°. The data is wirelessly transmitted to a mobile terminal via Bluetooth. This application demonstrates the potential of NB-Fiber in real-time gesture monitoring and recognition.

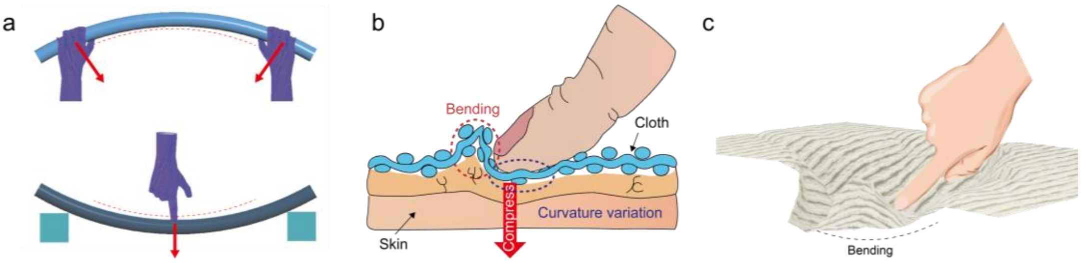


**Supplementary Fig. 29 Hand press on fabric induces curvature change illustration. a** Two methods of inducing curvature. **b** Localized pressing causes changes in fabric curvature; interference from pressure is challenging to eliminate when support is present. **c** Without support, the signal can be directly attributed to curvature. Bending involves a distributed force applied across the entire length of the fiber, resulting in curvature changes. On the other hand, pressing applies localized force, which also induces significant changes in curvature, extending beyond the conventional interpretation of bending.

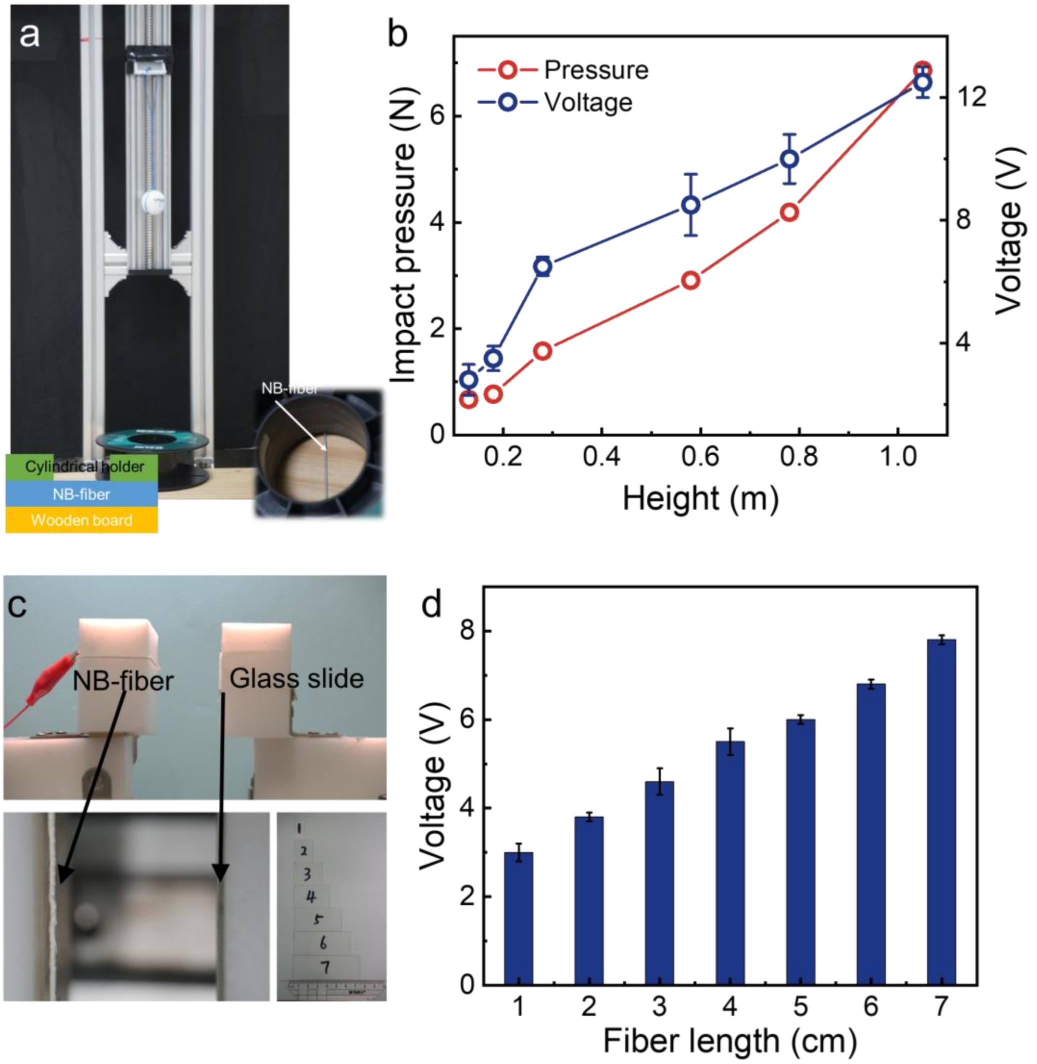


**Supplementary Fig. 30 The impact of different pressures and contact areas on NB-fiber triboelectric voltage. a** Utilizing free-falling golf balls to apply varying pressures. **b** The

impact of pressure on triboelectric voltage. **c** Employing glass pieces of different lengths to provide varying contact areas. **d** The influence of different contact areas on triboelectric voltage.

To address the pressing scenario further, we considered two cases illustrated in Supplementary Fig. 28b and c. In Supplementary Fig. 28c, where the fabric is without a supporting substrate, the contact and separation of the NB-fiber shell can be attributed to bending. However, when the fabric has a supporting substrate, as shown in Supplementary Fig. 28b, the influence of pressure becomes undeniable. We have conducted experiments to examine the effect of pressure magnitude and contact area on the triboelectric voltage of the NB-fiber with a supporting substrate (Supplementary Fig. 29).

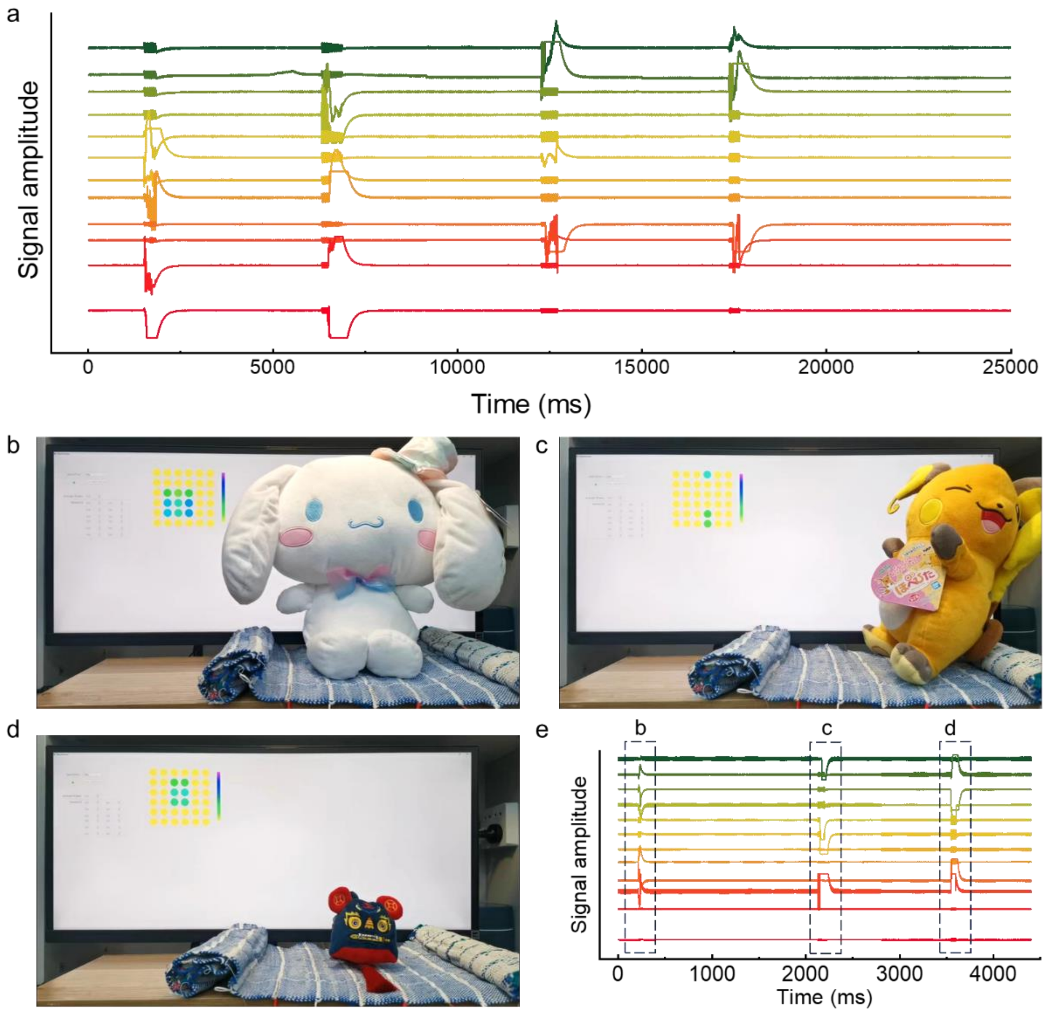


**Supplementary Fig. 31 Pressure mapping of various gestures and different doll sizes on NB-Textile. a** Data sets illustrating different gestures performed on NB-textile. **b** Pressure mapping when a doll with a large base is placed on NB-textile. **c** Pressure mapping when a doll

with a small base is placed on NB-textile. **d** Pressure mapping when a doll with a medium base is placed on NB-textile. **e** Corresponding sensor signal curves.

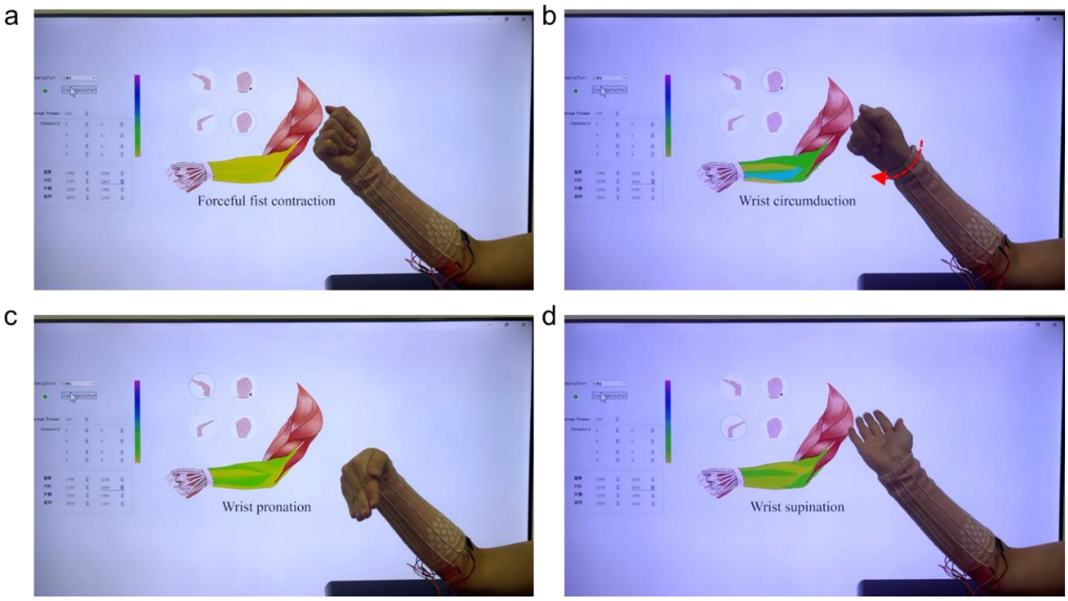


**Supplementary Fig. 32 Demonstration of muscle force mapping software under different hand gesture states. a** Fist gesture. **b** Wrist rotation. **c** Wrist pronation. **d** Wrist supination.

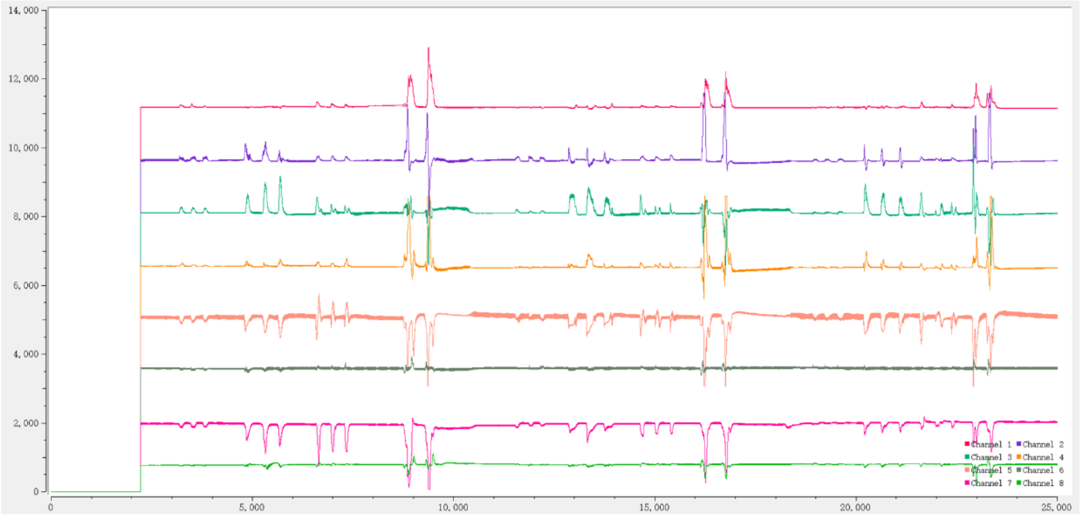


**Supplementary Fig. 33 The dataset of different hand gesture actions demonstrates the repeatability and stability of the monitoring system.**

**Supplementary** **Table 1** Compilation of literature on triboelectric systems for biomechanical bending sensing.

| No. | Refs. | Raw Data | Min. Bending Angle | Bending Accuracy | Flexure Factor |
|---|---|---|---|---|---|
| 1 | Nat Commun 13, 5224 (2022). | 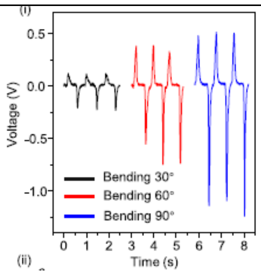 | 30° | 30° | ~6.7% |
| 2 | Nat Commun 12, 5378 (2021) | 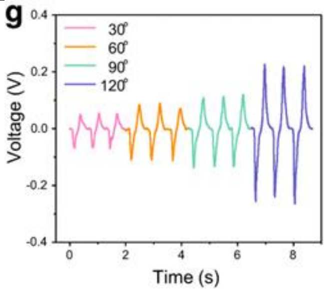 | 30° | 30° | ~2.8% |
| 3 | Sci Adv 6, eaba9624 (2020). | 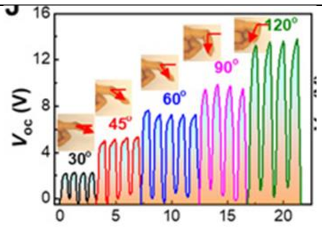 | 30° | 15° | ~4.1% |
| 4 | Sci Adv 6, eaaz8693 (2020). | 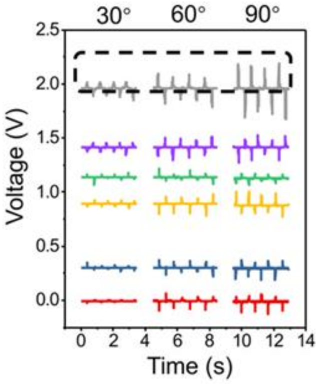 | 30° | 30° | ~3.3% |
| 5 | Sci Adv 6, eabb4246 (2020). | 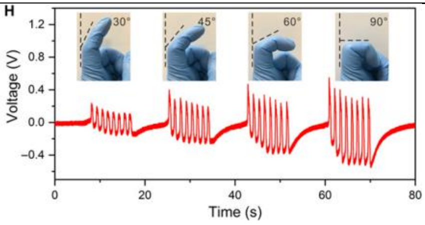 | 30° | 15° | ~2.8% |

| | | | | | |
|---|---|---|---|---|---|
| 6 | Nat Electron 3, 571–578 (2020). | 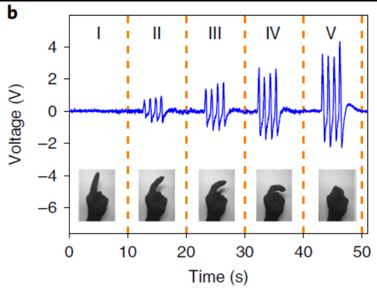 | ~30° | ~15° | ~5% |
| 7 | Our work | 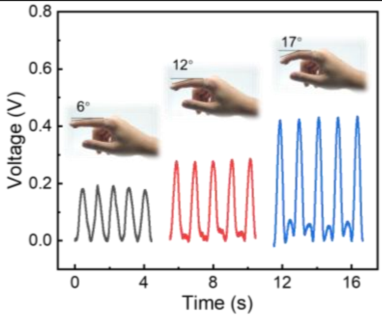 | ~6° | ~6° | 12.1% |

**Supplementary** **Table 2** Summary of testing details and data results from the cited reference.

| No. | Ref. | Test Details |
|---|---|---|
| 47 | Sci. Adv.4, eaaq0118 (2018). | 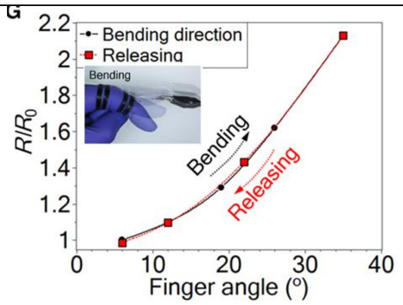 |
| 48 | Nat Commun 12, 3211 (2021). | 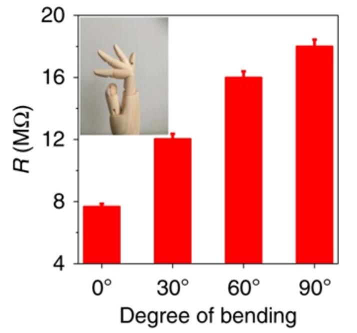 |
| 49 | Adv. Mater. 28, 722-728 (2016). | 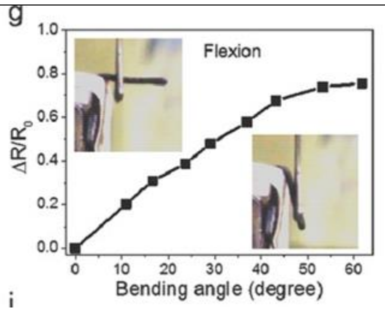 |
| 50 | Sci Adv 6, eabb4246 (2020). | 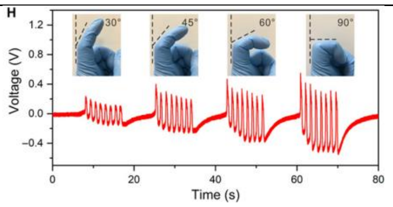 |
| ✧ | This work | 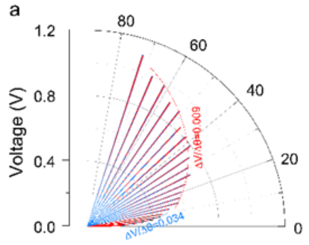 |

**Supplementary Table 3** Summary of strain sensing yarns: sensing mechanisms, electrical output, scalable manufacturing, recycling, and applications.

| **Refs.** | **Sensing mechanism** | **Device morphology** | **Electrical outputs** | **Scalable manufacture** | **stretchable** | **Robustness** | **Applications** |
|---|---|---|---|---|---|---|---|
| **Our Work** | Triboelectric effect | Fiber (nanofiber buckling structure) with nanostructure and large-size gap. | Internal self-generation: (L= 5 cm; 1 Hz): **0.9 V at curvature 6.0 $mm^{-1}$** | Industrial-level, Hundreds of meters | 100% reversible strain | 10000 | (1) Pulse, respiration, muscular effort; (2) Pressure space mapping; (3) Human acupoint pressure drawing; (4) Forearm muscle force monitoring and action recognition |
| 1 | Triboelectric effect | Fiber (built-in spiral structure) without nanostructure, and gap change is not evident during flexure. | Internal self-generation: (L= 5 cm; 1 Hz): **0.32 V at curvature 6.0 $mm^{-1}$** | Industrial-level, Hundreds of meters | <5% reversible strain | 2000 | Knee motion signal capture; |
| 2 | Triboelectric effect | Fiber (Fermat spiral structure) with nanostructure, and negligible gap | Internal self-generation: (L= 5 cm; 1 Hz): **no significant signal at a curvature of 6 $mm^{-1}$** | Industrial-level, Hundreds of meters | 100% reversible strain | 5000 | Gesture motion wireless sensor |
| 3 | Piezoresistive effect | Fiber (axial coating structure) without nanostructure and gap. | External power supply (L=5 cm): **$\Delta R/R_0$=5% at 90°** | Handmade | 1000% reversible strain | 600 | Wrist flex signal capture |
| 4 | Piezoresistive effect | Film (nanofiber network) | External power supply (1 × 2 $cm^2$): **$\Delta R/R_0$=100% at 45°** | Handmade | Not showed | 2000 | Gesture recognition |
| 5 | Piezoresistive effect | Fiber (spiral winding structure) without nanostructure, and negligible gap. | External power supply (L=3 cm): **$\Delta R/R_0$=80% at 60°** | Handmade | Not showed | Not showed | (1) Finger Movement Monitoring; (2) Pressure array mapping |
| 6 | Piezoresistive effect | Block (aerogels) | External power supply (1 × 2 × 0.1 $cm^2$): **$\Delta R/R_0$=10% at 90°** | Handmade | 50% reversible strain | 10000 | Finger bend test |
| 7 | Capacitive mechanism | Fiber (axial coating structure) without nanostructure, and negligible gap. | External power supply (L=3 cm): **$\Delta R/R_0$=25% at curvature of 60 $m^{-1}$** | Thermally Drawn, 10m | 100% reversible strain | Not showed | Not showed |

Note: Robustness in this table is assessed based on cycle life (unit: 1)


[1] Gong, W. et al. Nat. Commun. **10**, 868 (2019).
[2] Zhang, D. et al. *Adv. Mater*.**33**, e2100782 (2021).
[3] Zheng, L. et al. *Sci. Adv*. **7**, eabg4041 (2021).

[4] Wang, B. et al. *Nat. Commun*. **11**, 2405 (2020).
[5] Ge, J. et al. *Adv. Mater.* **28**, 722-728 (2016).
[6] Min, P. et al. *Adv. Funct. Mater.* **31**, 2103703 (2021).
[7] Leber A. et al. *Adv. Sci.* **10**, 2207573 (2023).

## Supplementary Notes

### Supplementary Note 1 Regulating the amplitude of fiber wrinkling in the model and its correlation with flexure sensitivity.

We fine-tuned the model by analyzing the deformation energy levels within the substrate and nanofiber network to determine the dimensionless parameter Cs.

$$C_s = \frac{E_s}{E_f}(\frac{R}{h_f})^{3/2} \tag{1}$$

Where the elastic moduli of the core and shell are represented by Es and Ef, respectively. R represents the radius of the core, and hf signifies the thickness of the sheath. It becomes apparent that when axial prestress is released in the cylindrical core-shell structure, increasing R, the nanofiber network shell tends to buckle into a sinusoidal mode. As the internal shape memory material undergoes thermal recovery, the radial radius R of the core contracts, creating gaps between layers that promote subcritical secondary bifurcation instability within the cylindrical sheath-core structure. This instability mode induces buckling of the nanofibrous network on the surface, forming an oriented wrinkled cavity structure called secondary twist. The secondary twist further enhances the dynamic mechanical stability of the yarn.

To visualize the high-resolution voltage signal resolution of the nanofiber bucking structure, we utilized COMSOL Multiphysics (Supplementary Fig. 1) to characterize the relationship between voltage U and the interlayer distance h using the capacitance model.

$$U(h) = \frac{\sigma h}{\varepsilon_0} \tag{2}$$

In the capacitance model, σ represents the surface charge density, and $\varepsilon_0$ is the dielectric constant of the interlayer dielectric. Increasing the interlayer distance leads to a higher gap potential difference.

By assuming plane strain in the cylindrical core-shell structure and simplifying the circular plane strain model, we can obtain the wrinkling amplitude A and critical strain $\varepsilon_c$.

$$\varepsilon_c = \sqrt{\frac{E_s h_f}{3RE_f}},\ A = h_f\sqrt{\frac{2}{3}}(\frac{\varepsilon}{\varepsilon_c} - 1) \tag{3}$$

The formula indicates that reducing the substrate modulus Es can increase the wrinkling amplitude, corresponding to the interlayer spacing h. Furthermore, Supplementary Fig. 3 demonstrates the relationship between the material's flexural modulus and its type and shape. Hence, to further enhance the voltage resolution of NB-structure, we opted to construct the core using an EVA shape memory tube.

For simplicity, we focus on the moment when the nanofiber network shell is in complete contact with the core to investigate the relationship between the dynamically variable interlayer spacing

of NB-structure and flexure sensitivity. By observing the constant arc length before and after bending. We derive the following Equation:

$$h = \frac{\sqrt{(1-\frac{(r+R+h_f)\theta}{2n^2})^2-1}}{n} \quad (4)$$

In this Equation, r represents the curvature radius of the system, n denotes the wave number of the nanofiber network shell, and θ is the bending angle. Moreover, it is evident from the Equation that Δθ is positively correlated with Δh, further illustrating that increasing the dynamic variable layer spacing Δh can enhance the voltage resolution of the yarn.

**Supplementary Note 2 Synthesis of ionogel electrodes.**

Copolymer ionogels were synthesized via a one-step method through random AAm and AA monomers copolymerization. In the standard procedure, a homogeneous solution was prepared by dissolving these two monomers in 3.3 ml of 1-Butyl-3-methylimidazolium tetrafluoroborate, maintaining a prescribed total concentration ($C_m$ = 6 M) and a molar fraction of AAm (x = 0.8). To this solution, the covalent cross-linker N,N'-methylenebis(acrylamide) was incorporated at a concentration of 0.1 mol% relative to $C_m$, in conjunction with the thermal initiator ammonium persulfate (0.1 mol% relative to $C_m$). The resulting solution was cast into a mold, where a spacer spatially separated two glass pieces and subsequently subjected to heating at 60 °C for 1 hour to facilitate the formation of the copolymer ionogel.

**Supplementary Note 3 Comprehensive evaluation of flexure performance using curvature.**

The radius of curvature (R) was defined as the ratio of the change in arc length (dL) to the change in tangential angle (dα) along a fixed point on the curve. In contrast to conventional methods employed in triboelectric nanogenerators that primarily rely on output voltage amplitude to quantify flexure angle, we comprehensively assessed the flexure performance by considering both the degree of curvature and the fiber length. Supplementary Fig. 11 illustrates the results of the NB-fiber length, angle, and frequency analysis. This approach ensures a rigorous and professional evaluation of the bending characteristics in our study.

**Supplementary Note 4 Analyzing the reasons for insensitivity to bending in fermat spiral spinning and built-in spiral.**

Specifically, the Fermat spiral spinning technology provides a paradigm for long-range ordered assembly of nanofiber networks, exploiting the inhomogeneous bonding of two-phase materials on a rough surface to achieve in situ shaping of interfacial gaps. However, the gap (typically

between 0–20 μm) is minimal due to simple mechanisms' limited stress storage-release capability. As a result, the potential change of the fiber is negligible under subtle topological transitions, and the sensitivity is insufficient under micro-flexure (yielding no signal even under 100° flexure). The scalability and versatility of thermal drawing provide another idea for the continuous construction of a large gap by prefabricated hollow structures, using the difference in Young's modulus of the two-phase materials to adjust the spinning stress and realize the large cavity with a built-in spiral. However, due to the similar flexural moduli of the two-phase materials, the curved surface restricts the topological deformation of the inner electrodes. Therefore, the large cavity created by this artificially prefabricated hollow structure does not change significantly during flexure.

**Supplementary Note 5 Flexure factor: defining and quantifying bending sensitivity.**

Flexure sensitivity refers to the capacity of a material or device to detect and respond to flexural deformations. It is quantified using the flexure factor, which measures the relative change in output signal amplitude under identical flexure stimuli. The flexure factor is computed by comparing the output signal amplitude in response to a specific flexure stimulus with the baseline or initial signal amplitude. This factor is defined as the ratio of the relative amplitude of the output to the difference in bending angles, expressed as a percentage.

$$Flexure\ Factor = \frac{Relative\ Amplitude}{Difference\ in\ Bending\ Angles} \times 100\%$$

This factor provides a quantitative assessment of the material or device's sensitivity to flexure, indicating its ability to detect and convert flexure deformations into measurable signals. Precise calculation and interpretation of the flexure factor enable accurate comparisons and evaluations of the flexure performance across different sensing systems or designs.

**Supplementary Note 6 Calculation method and analysis of augmentation index.**

The augmentation index (AIx) is a widely utilized parameter for analyzing arterial pulse waveforms to evaluate arterial stiffness and wave reflection, providing valuable insights into the hemodynamic characteristics of the human body. The accurate calculation and analysis of AIx require specific methods and parameters. The calculation process involves the following steps: (1) Acquiring the pulse waveform using high-fidelity pressure sensors or tonometers from peripheral arteries, such as the radial artery. (2) Processing the acquired pulse waveform to identify critical features, including the systolic peak (P1), diastolic peak (P2), and inflection point (P3). (3) Determining the augmentation pressure (AP), which represents the contribution of wave reflection to the central aortic pressure, by calculating the difference between the second systolic peak (P2) and the inflection point (P3) on the pulse waveform. (4) Calculating

the AIx as a percentage using the formula AIx (%) = (AP / Pulse Pressure) * 100, where Pulse Pressure is the difference between the first and second systolic peaks (P1 - P2) on the pulse waveform. AIx is influenced by age, heart rate, blood pressure, and measurement location. The accurate calculation and analysis of AIx offer valuable insights into cardiovascular health and disease.
