# Supplementary material for: Triboelectric micro-flexure-sensitive fiber electronics
Source: Nat Commun. 2024 Mar 15;15:2374. doi: 10.1038/s41467-024-46516-0 (PMC10943239; doi:10.1038/s41467-024-46516-0)
Supplement: Supplementary file 3 — Description of Additional Supplementary Files [file 41467_2024_46516_MOESM3_ESM.pdf]

<u>Description of Additional Supplementary Files</u>

<u>Supplementary Movie Legends:</u>

File Name: Supplementary Movie 1

Description: Continuous and scalable manufacture of micro-flexuresensitive fiber enabled by nanofiber buckling (NB-fiber) with dynamic stability structural.

File Name: Supplementary Movie 2

Description: Prospects of NB-fiber in subtle physiological diagnosis.

File Name: Supplementary Movie 3

Description: Application of NB-textile in dynamic path recognition.

File Name: Supplementary Movie 4

Description: Application of NB-textile in human acupoint pressure mapping.

File Name: Supplementary Movie 5

Description: Application of NB-textile in forearm muscle force monitoring and motion recognition.
