# Supplementary material for: Triboelectric micro-flexure-sensitive fiber electronics
Source: Nat Commun. 2024 Mar 15;15:2374. doi: 10.1038/s41467-024-46516-0 (PMC10943239; doi:10.1038/s41467-024-46516-0)
Supplement: Supplementary file 9 — Reporting Summary [file 41467_2024_46516_MOESM9_ESM.pdf]

nature portfolio

Corresponding author(s): Hongzhi Wang

Last updated by author(s): Feb 2, 2024

Reporting Summary

Nature Portfolio wishes to improve the reproducibility of the work that we publish. This form provides structure for consistency and transparency in reporting. For further information on Nature Portfolio policies, see our Editorial Policies and the Editorial Policy Checklist.

Statistics

For all statistical analyses, confirm that the following items are present in the figure legend, table legend, main text, or Methods section.

n/a	Confirmed	
☐	☒	The exact sample size (*n*) for each experimental group/condition, given as a discrete number and unit of measurement
☐	☒	A statement on whether measurements were taken from distinct samples or whether the same sample was measured repeatedly
☒	☐	The statistical test(s) used AND whether they are one- or two-sided<br>*Only common tests should be described solely by name; describe more complex techniques in the Methods section.*
☒	☐	A description of all covariates tested
☒	☐	A description of any assumptions or corrections, such as tests of normality and adjustment for multiple comparisons
☐	☒	A full description of the statistical parameters including central tendency (e.g. means) or other basic estimates (e.g. regression coefficient) AND variation (e.g. standard deviation) or associated estimates of uncertainty (e.g. confidence intervals)
☒	☐	For null hypothesis testing, the test statistic (e.g. *F*, *t*, *r*) with confidence intervals, effect sizes, degrees of freedom and *P* value noted<br>*Give P values as exact values whenever suitable.*
☒	☐	For Bayesian analysis, information on the choice of priors and Markov chain Monte Carlo settings
☒	☐	For hierarchical and complex designs, identification of the appropriate level for tests and full reporting of outcomes
☒	☐	Estimates of effect sizes (e.g. Cohen's *d*, Pearson's *r*), indicating how they were calculated

Our web collection on statistics for biologists contains articles on many of the points above.

Software and code

Policy information about availability of computer code

Data collection	Keithley 6514E and a professional acquisition program were employed to capture human respiratory and pulse signals. Additionally, a custom-developed electronic module and commands were utilized to capture signals related to acupressure on human acupuncture points and muscle deformation.
Data analysis	The plots in this manuscript were generated using Origin 2021, and no custom data analysis was performed.

For manuscripts utilizing custom algorithms or software that are central to the research but not yet described in published literature, software must be made available to editors and reviewers. We strongly encourage code deposition in a community repository (e.g. GitHub). See the Nature Portfolio guidelines for submitting code & software for further information.

Data

Policy information about availability of data

All manuscripts must include a data availability statement. This statement should provide the following information, where applicable:

- Accession codes, unique identifiers, or web links for publicly available datasets
- A description of any restrictions on data availability
- For clinical datasets or third party data, please ensure that the statement adheres to our policy

Provide your data availability statement here.

Research involving human participants, their data, or biological material

Policy information about studies with human participants or human data. See also policy information about sex, gender (identity/presentation), and sexual orientation and race, ethnicity and racism.

Reporting on sex and gender	Our study is not focused on gender-related aspects, and we have not collected specific sex or gender data in our research. Therefore, we confirm that the findings of our study are not related to sex or gender, and we did not perform sex- or gender-based analyses.
Reporting on race, ethnicity, or other socially relevant groupings	The study focused on other aspects, and no information related to race, ethnicity, or other socially relevant groupings was collected or analyzed. As a result, there were no confounding variables related to these factors that needed to be controlled in our analyses.
Population characteristics	Five healthy participants, aged between 20 and 60 years, consisting of 2 females and 3 males.
Recruitment	The participants were recruited internally from within the research group. We provided a detailed description of the study, explicitly stating that participation was voluntary and non-participation would have no impact.
Ethics oversight	The study protocol received approval from the Ethics Review Board of Donghua University, and the human-related experiments were conducted in accordance with the guidelines outlined in the testing protocol (Protocol Number: DHUEC-NSFC-2022-43, Protocol Title: Sensitivity Testing of Fibrous Electronic Devices). No specific stimuli posing potential harm to humans were applied; instead, the sensor fibers were tested by placing on the body surface.

Note that full information on the approval of the study protocol must also be provided in the manuscript.

Field-specific reporting

Please select the one below that is the best fit for your research. If you are not sure, read the appropriate sections before making your selection.

☒ Life sciences ☐ Behavioural & social sciences ☐ Ecological, evolutionary & environmental sciences

For a reference copy of the document with all sections, see nature.com/documents/nr-reporting-summary-flat.pdf

Life sciences study design

All studies must disclose on these points even when the disclosure is negative.

Sample size	Three male and two female volunteers, all recruited voluntarily. The dataset includes two video segments and multiple images.
Data exclusions	No data excluded.
Replication	Different devices made with the same materials were tested for multiple times with different activities.
Randomization	Randomization was not relevant to our study since experiments were specific to each subject.
Blinding	Blinding was not relevant to our experiments as there were no subjective elements to assessing the device's capabilities.

Reporting for specific materials, systems and methods

We require information from authors about some types of materials, experimental systems and methods used in many studies. Here, indicate whether each material, system or method listed is relevant to your study. If you are not sure if a list item applies to your research, read the appropriate section before selecting a response.

Materials & experimental systems

n/a	Involved in the study	
☒	☐	Antibodies
☒	☐	Eukaryotic cell lines
☒	☐	Palaeontology and archaeology
☒	☐	Animals and other organisms
☒	☐	Clinical data
☒	☐	Dual use research of concern
☒	☐	Plants

Methods

n/a	Involved in the study	
☒	☐	ChIP-seq
☒	☐	Flow cytometry
☒	☐	MRI-based neuroimaging

Plants

Seed stocks

Report on the source of all seed stocks or other plant material used. If applicable, state the seed stock centre and catalogue number. If plant specimens were collected from the field, describe the collection location, date and sampling procedures.

Novel plant genotypes

Describe the methods by which all novel plant genotypes were produced. This includes those generated by transgenic approaches, gene editing, chemical/radiation-based mutagenesis and hybridization. For transgenic lines, describe the transformation method, the number of independent lines analyzed and the generation upon which experiments were performed. For gene-edited lines, describe the editor used, the endogenous sequence targeted for editing, the targeting guide RNA sequence (if applicable) and how the editor was applied.

Authentication

Describe any authentication procedures for each seed stock used or novel genotype generated. Describe any experiments used to assess the effect of a mutation and, where applicable, how potential secondary effects (e.g. second site T-DNA insertions, mosiacism, off-target gene editing) were examined.
